# Creation and validation of the Picture-Set of Young Children’s Affective Facial Expressions (PSYCAFE)

**DOI:** 10.1371/journal.pone.0260871

**Published:** 2021-12-07

**Authors:** Matthias Franz, Tobias Müller, Sina Hahn, Daniel Lundqvist, Dirk Rampoldt, Jan-Frederik Westermann, Marc A. Nordmann, Ralf Schäfer

**Affiliations:** 1 Medical Faculty, Clinical Institute for Psychosomatic Medicine and Psychotherapy (15.16), University Hospital Düsseldorf, Düsseldorf, Germany; 2 Karolinska Institute, Department of Clinical Neuroscience, NatMEG, Solna, Sweden; National Institutes of Health, UNITED STATES

## Abstract

The immediate detection and correct processing of affective facial expressions are one of the most important competences in social interaction and thus a main subject in emotion and affect research. Generally, studies in these research domains, use pictures of adults who display affective facial expressions as experimental stimuli. However, for studies investigating developmental psychology and attachment behaviour it is necessary to use age-matched stimuli, where it is children that display affective expressions. PSYCAFE represents a newly developed picture-set of children’s faces. It includes reference portraits of girls and boys aged 4 to 6 years averaged digitally from different individual pictures, that were categorized to six basic affects (fear, disgust, happiness, sadness, anger and surprise) plus a neutral facial expression by cluster analysis. This procedure led to deindividualized and affect prototypical portraits. Individual affect expressive portraits of adults from an already validated picture-set (KDEF) were used in a similar way to create affect prototypical images also of adults. The stimulus set has been validated on human observers and entail emotion recognition accuracy rates and scores for intensity, authenticity and likeability ratings of the specific affect displayed. Moreover, the stimuli have also been characterized by the iMotions Facial Expression Analysis Module, providing additional data on probability values representing the likelihood that the stimuli depict the expected affect. Finally, the validation data from human observers and iMotions are compared to data on facial mimicry of healthy adults in response to these portraits, measured by facial EMG (m. zygomaticus major and m. corrugator supercilii).

## 1. Introduction

It is a crucial ability of human interaction to detect and to understand affective signals, to represent them verbally and to display them in direct social contact. An impairment of this ability is often associated with various psychic or psychosomatic disorders [1–3]. Although these disorders are heterogenous with regard to ethology and symptomatology, all of them are characterized by deficits in affect recognition and processing.

To assess such deficits reliably, there is a need for validated affective stimuli consisting of facial expressions that represent the basic affects. Additionally, from an evolutionary point of view, affect expressive facial mimic and mimicry may have a different meaning and communicative function in different phases of life. In early childhood, survival and development of attachment are based on the affect-related regulation of the needs of the child by the primary caregiver. This process is stimulated by the baby schema [4]. Affect expressive mimic in adulthood is an important carrier of emotional information for conflict regulation [5] and mate selection [6]. Because of that, it is vital to create picture-sets of affect expressive faces for different age groups. This allows to investigate affect recognition and processing without the confounding effect of the own-age-bias [7] or to perform studies on attachment related and developmental issues such as early parent-child-interaction and affect regulation (e.g. dependent from different psychic or affective disorders).

### 1.1 A brief history of facial emotional stimuli

In 1982 Ekman established the term of basic emotions. Each of these basic emotions can be described by means of a specific affect, related physiological reactions, certain intentions to act, and a specific facial expression. These universal basic emotions include fear, disgust, happiness, sadness, surprise and anger [8, 9].

Based on their studies, Ekman & Friesen developed a first picture-set consisting of portraits of adults who display the six basic affects [10]. The increased interest in research on facial affect detection and reaction to facial affect signals led to a large number of picture-sets, which assess these abilities. Studies on facial mimicry—the unconscious imitation of facial affect expressions—can be given as an example. Studies that examined the variety of mimic reaction to affective faces [11, 12] and other factors influencing facial mimicry [13–15] most commonly used picture-sets of affect expressive portraits as stimulus material. In addition, further picture-sets were generated which show individuals of different age groups, different ethnic backgrounds or being displayed in varying perspectives (e.g. The Karolinska directed emotional faces (KDEF) [16]; A lifespan database of adult facial stimuli [17]; The NimStim Set of Facial Expressions [18]; FACES—A database of facial expressions in young, middle-aged, and older women and men [19]; The NIMH Child Emotional Faces Picture Set (NIMH-ChEFS) [20]; The Developmental Emotional Faces Stimulus Set (DEFSS) [21]. While a wide range of picture-sets of adults and adolescents is currently available, only a small number of picture-sets presenting affective children’s faces exist (e.g. The Dartmouth Database of Children’s Faces [22]; The Child Affective Facial Expression–CAFE [23]; The EU-Emotion Stimulus Set [24]; The Tromso Infant Faces Database (TIF) [25]. Contrary to the set described here, CAFE, The Dartmouth Database of Children’s Faces and The EU-Emotion Stimulus Set do not include averages of prototypical deindividualized facial expressions created in digital format, but individual pictures of real persons that can be clearly identified.

### 1.2 Why PSYCAFE?

Concerning scientific research in the fields of attachment behaviour or the development of affect-processing, it is crucial to have a validated picture-set of young children available. Based on a pilot study [26] a new picture-set was developed in the course of this study consisting of digitally deindividualized affect prototypical portraits of children aged 4 to 6 years. Rampoldt developed a concept for theatre workshops in order to teach young children to get to know the characteristics of different affects and their mimic expressions. 22 children (15 female and 7 male) took part in these workshops. Their facial expressions of the different affects were filmed. 90 single frame images were gained out of this video material; they were validated by 220 subjects regarding intensity of the facial expression, its authenticity and likeability. The final selection of pictures that could be assigned to one affect was conducted by use of a hierarchical cluster analysis and the Facial Action Coding System [27].

The unique character of the PSYCAFE picture-set can be explained by the protagonists’ age range and the quality of the pictures. The faces of young children show certain characteristics that can be summed up by the term baby schema [28]. This principle of infantile facial features influences both attachment affinity of adults and the child’s own affective development [4]. The adult’s increased readiness for attention and mirroring, triggered by the baby schema, results in an evolutionary survival benefit for the child [29]. Therefore, a picture-set of affect expressive faces of children could be used to further investigate effects of the baby schema on the emotional processing of affects and to identify differences from the processing of adult affect expressive faces. In particular, this picture-set could be of considerable use in the study of clinical samples in which the parent-child relationship plays a major role, such as postpartum depression, or e.g. within experimental studies on attachment.

Usually, picture-sets of affective facial expression comprise individual portraits of real persons [18, 20, 23], see however [30], whereas the picture-set presented in this study consists of deindividualized and prototypical affective portraits.

These were generated by means of digital addition using several individual portraits of real children within a certain range of age. There are two important reasons for creating a picture-set of affect prototypical deindividualized portraits of children. Firstly, there is an ethical argument to deindividualize pictures of children. A toddler is not able to give its informed consent to spread its individual face irreversibly in the public. In no case, a later harm can be excluded. As an adult, the former child may be harmed by the unwanted widespread visibility of his or her publicized infantile face. This could be a violation of personal rights resulting in possible emotional distress for the individual.

The other argument addresses methodological aspects. Morphological characteristics of the individual face may confound and bias the mimic affect recognition depending on personal experiences and preferences of the observer [31]. Prototypical expressions abstract from the individual variation in craniofacial properties and capture the between-individual commonalities in expressions. In addition, the performance to display facial affects differs between individuals. This is true particularly for young children who are not able to follow instructions very precisely. Thus, observer ratings in validation studies show remarkable scatter [32]. While Ekman and Cordaro [33] stated that basic affects are expressed universally among humans, studies have shown that the reliability of affect detection differs depending on the individual face [19, 32, 34]. Therefore, to purify the affect signal and to rule out the influence of individual morphological details and variance in affect expression, deindividualized affect prototypical pictures averaged from validated individual portraits may be a reasonable choice.

This allows to present prototypical features of affective facial expression while the individual child cannot be identified. Furthermore, the visibility of specific facial features or the peculiarities of the individual affective expression can be avoided by means of averaging. Thus, for a distinct facial expression, prototypical reference pictures were digitally created from children’s portraits by using pictures of both a basic affect and gender.

This study representing the development of a new set of affect expressive portraits of children is structured in four steps: (1) the creation of individual affect expressive portraits of preschool children within a naturalistic environment, (2) the selection and validation of these individual portraits according to six basic affects, (3) the computing of deindividualized prototypical affect expressive facial mimic by digital overlay and averaging the individual portraits of these children, (4) the validation of these averaged faces, (5) step 3 and 4 are also applied for those affect expressive portraits of an existing set of affect expressive facial mimic of adults (KDEF), which were classified as valid by observer ratings [34].

This study investigates both digitalized picture-sets regarding the intensity of the different facial expressions, the authenticity, the likeability for the presented picture and the percentage of participants having correctly identified the presented affect.

Regarding authenticity, there are several studies investigating differences between posed and genuine facial expressions [35]. The aim of this study was to create authentic pictures. Therefore, we examined whether our picture-set of children gained similar or even higher ratings in authenticity in comparison to the adult pictures that were created with trained amateur actors.

Furthermore, we examined participants’ response to the portraits in terms of likeability in order to draw conclusions about perceived affection and to examine effects of the baby schema. Due to the effect of the baby schema and adults’ attachment behaviour, it is supposed that the likeability for children is rated higher than for the pictures of adults. In this case, an increased likeability of affect expressive faces in children compared to adults could bias the assessment (decreased) and physiological reactions (diminished) in the processing of aversive facial expressions [36, 37].

Furthermore, the hit-rates respective the affect recognition performance of observers were investigated as a validation criterion of the stimulus material. Additionally, the affect detection probability provided by a facial affect recognition software (AFFDEX from iMotions) for each displayed affect and its comparison to the electromyographically measured facial reaction (m. zygomaticus and m. corrugator supercilii) of healthy adults to these stimuli [37] serves as a further aspect of validity.

Finally, the goal of this study is to generate a valid naturalistic but deindividualized and affect prototypical picture-set of facial portraits of preschool aged children. This tool may facilitate research in the field of age dependent affect perception or parental competences in affect regulation e.g. dependent from attachment style or psychic impairment.

**Fig 1:**
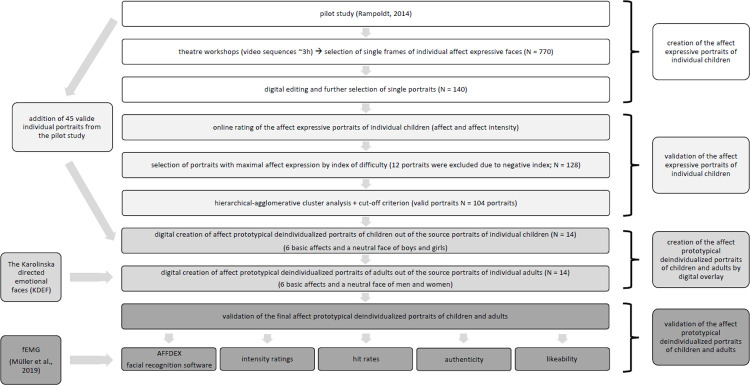
Procedure of the present study.

A precise schedule of the study including the different steps of the statistical analyses is shown in Fig 1.

## 2. Materials and methods

### 2.1 Creation of the affect expressive portraits of children

In a first step, individual affect expressive portraits of children were created in the context of three theatre workshops. A drama teacher in three different municipal day-care centres conducted these workshops, specifically designed for children. The participants were recruited through the distribution of information sheets. 35 children (14 male, 21 female) within the age range from 4 to 6 years participated in these workshops. Both the children and their parents received detailed information about the study process in written and verbal form. Parents gave their written informed consent for the child’s participation in the study.

By taking part in an interactive designed adventure trip, the children became familiar with the characteristics of the six basic affects and were able to express them in a child-friendly way. In the course of this game, each child took place on a chair in front of a white screen in an adjacent room and was filmed from a frontal perspective (Canon Legria HF S 200E).

Specific anatomic muscle-by-muscle instructions are difficult to realize working with preschool children. In analogy to the Directed Facial Action Task [38], children were asked to emphasize different facial expressions by moving anatomical landmarks (e.g. eyebrows). Furthermore, they were encouraged to remember situations in which they experienced the specific affect. The video recording of each child took 5 to 8 minutes.

The frame representing the maximal expression of the six basic affects (apex) within 770 video sequences was identified by one of two experienced raters per child and saved as a still image. Due to several criteria (intensity of the facial affect expression, image sharpness, rotation of the face, closed eyes, illumination etc.), 630 portraits were excluded from the 770 selected pictures. The remaining 140 pictures were then digitally (Adobe Photoshop Pro CS 6) edited (adjustment of the contrasts, adapting the picture’s size).

### 2.2 Validation of the affect expressive portraits of children

In a second step, the 140 individual affect expressive portraits of children were validated in an online rating in order to identify those portraits that could be clearly assigned to a single affect. Participants were recruited via social media as well as announcements for the study on the Internet. This validation involved several steps using an internet portal for online rating (Unipark; Questback). Using the website www.random.org, the 140 pictures were randomly and equally distributed to two groups by affect and gender with 70 pictures per group. Thus, the workload of the online rating was reduced for the raters, and the participants were more likely to complete the survey. In the anonymous survey, each participant received an individual link that limited to one-time participation. Subsequently, the individual code of the link expired to guarantee that the links were not passed on to a third party, and to prevent unauthorized persons to gain access to the pictures.

The participants had to read an information sheet first to give their consent to participate in the study by ticking a box. Then they were randomized to one of the two groups by the software Unipark. Personal data of the participants (gender, age and level of educational qualification, the number of own children and the frequency of contact to children in their daily lives) were collected. Subsequently, the participants rated the 70 pictures of each group with respect to the dimensions *affect intensity*, *authenticity* and *likeability*.

The 70 affect expressive and neutral pictures were presented in random order without any time limit. Each portrait was depicted on a separate page and the participants were asked to specify the displayed affect of the children’s face. Answering options were the six basic affects (fear, disgust, happiness, sadness, anger, surprise) and a neutral facial expression. Multiple answers were possible to ensure a precise description of the perceived affects. There was no choice “no answer”, instead the participants could proceed without selecting an affect. On the second page, the participants assessed the intensity of every affect they chose on a five-point Likert-scale (1 = very weak; 2 = weak, 3 = moderate, 4 = strong, 5 = very strong). The authenticity of the expression and the perceived likeability towards the specific face were assessed likewise. Afterwards, the participants completed the German version of the Toronto Alexithymia Scale 20 (TAS-20), which is a common questionnaire to assess alexithymia, focusing on difficulties in identifying and describing feelings and the extent of externally oriented thinking [39, 40]. Thus, the sample could be examined with respect to the distribution of alexithymic subjects, since an accumulation of alexithymia and the associated difficulties in identifying feelings could have altered the results of the validation ratings.

The obtained data were analysed using SPSS 23 and Excel 2010. In a first step the two rater groups, each assessed 70 pictures, were tested on differences using t-tests for independent samples. There were no significant differences between the groups in age (*t*(195) = -1.28; *p* = .204) and the sum score of the TAS-20 (*t*(195) = 0.81; *p* = .418). By means of a chi-square-test, both groups were tested on equal distribution of gender. They did not differ (*Χ*^2^ = .25; *p* = .62; Cramér’s *V* = 0.036; *p* = .62) and therefore, both groups were combined for further analysis. The probability of an alpha error was 5% for all tests.

The entire rater sample included 197 participants (132 (67%) women and 65 (33%) men). The participant’s mean age was *M* = 32.9 years, (*SD* = 16.1) with a range from 18 to 88 years. 70% of the participants were younger than 30 years. Most of the participants had a university degree (47%) or a high school diploma (36%). The mean sum score of the TAS-20 was *M* = 43.6 (*SD* = 8.9). Only 4% of the sample had a sum score ≥ 61. This implies, according to epidemiological data, that there was a lower proportion of alexithymic participants in the sample than in the general population [40].

In order to analyse the evaluation of the pictures, the value of intensity of all portraits was calculated. A facial expression was assigned to a specific affect, if there was a combination of a high intensity-value of a specific affect and low intensity-values of the remaining affects. An index of difficulty was calculated for each picture. All participants assessed each portrait with respect to the intensity of all six basic affects on a Likert-scale between 0–5. The six overall means of intensity were calculated for each affect dimension. The five lowest intensity-values were subtracted from the affect with the highest rating. Pictures with an index higher than 0 were assigned to a specific affect. Portraits with index values below 0 were excluded from further analysis. Only portraits of neutral facial expressions should show low ratings of intensity and an index of difficulty around 0.

Afterwards, a hierarchical cluster analysis was performed in analogy to the pilot study as a further confirmatory validation step for the remaining pictures using the intensity ratings for all six basic affects as criterion. This made it possible to assign portraits to groups that were as homogeneous as possible and in a second step, to identify those clusters which could be clearly assigned to a single affect on the basis of the intensity ratings. The hierarchical agglomerative method was chosen as a grouping aggregation procedure [41]. Initially, each picture forms a distinct cluster, then the single clusters merge gradually together. By means of the cluster method average-linkage [41] the objects’ mean distance within the cluster was calculated and compared pairwise with the mean values of the other clusters. Those clusters with the shortest distance between each other were grouped together. The Euclidian distance was used as distance measure. This technique was applied in order to group pictures with similar patterns of intensity of facial expression.

The aim of the analysis was to assign all pictures to seven distinct clusters (six basic affects + neutral expression). By means of cluster analysis both the optimal number of clusters and the pictures’ assignment to the clusters were determined. If more than seven clusters were defined, the clusters, which did not meet a specific cut-off criterion, were eliminated.

To meet the criterion, the highest rated mean value of intensity of the portraits within a cluster had to receive a minimum of the value 3, and the second highest value of intensity had to achieve a value of a category being at least two categories below the highest intensity. Using the highest intensity rating for every cluster, the remaining clusters were aggregated to seven clusters (six basic affects, neutral face).

A confirmatory hierarchical cluster analysis by Ward [41] was used to verify the seven-cluster solution of the first cluster analysis. This method implies the aggregation of those clusters, which create the smallest growth of variance. The squared Euclidian distance was used as distance measure.

The described method was also applied on the clusters of the pilot study of Rampoldt [26]. All pictures of the pilot study fitting the cut-off criterion were included in the following creation of the affect prototypical reference portraits.

### 2.3 Creation of the affect prototypical deindividualized portraits of children and adults

**Fig 2:**
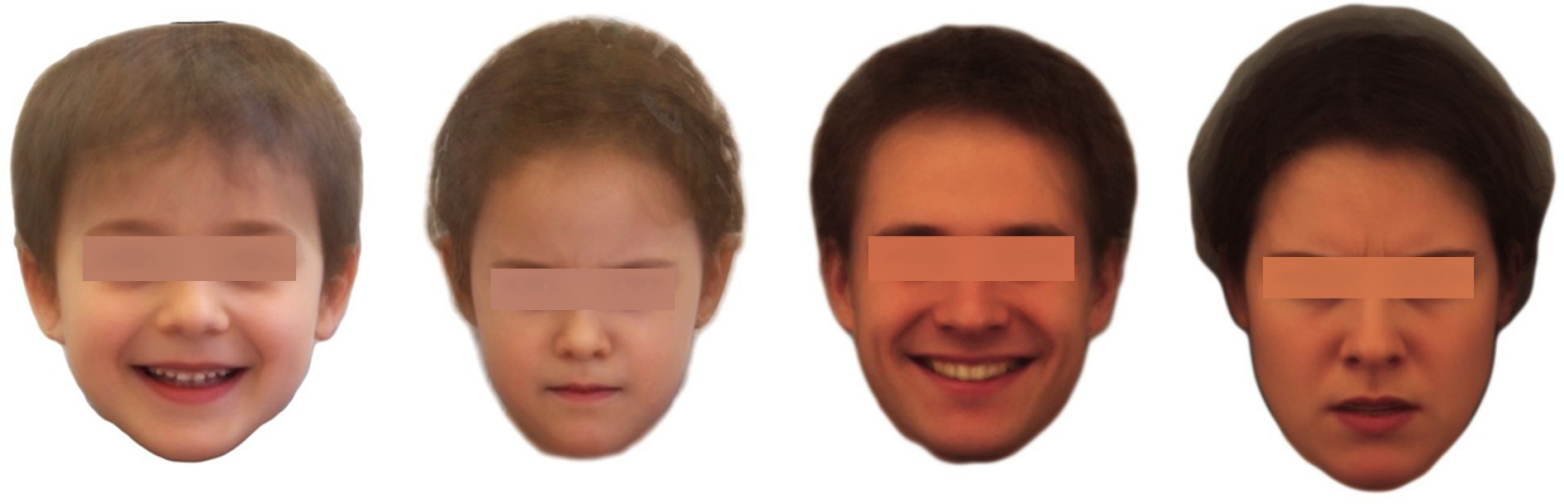
Examples of the 28 prototypical reference portraits of the basic affects happiness (child, male), anger (child, female), happiness (adult, male) and anger (adult, female) (from left to right).

Both the portraits from the pilot study and the individual pictures of children created in this study were used in a next step to create deindividualized affect prototypical average portraits using digital overlay. Individual portraits of adults from the already validated KDEF picture-set of the Karolinska institute [16] were used to create comparable deindividualized adult portraits in order to be able to investigate differences in affect processing with respect to the age of the depicted persons and the impact of the baby schema. This picture-set was already validated also by observer ratings [34]. The KDEF picture-set includes a high number of portraits of adults presenting the seven expressions (six basic affects and the neutral expression). KDEF was chosen because it is one of the most widely used and well validated stimulus materials for facial emotional expressions available. For each individual and expression, facial features were aligned to a template grid in the camera, so that key facial features (eyes and mouth) were always centred (horizontally) and found in the same spatial area (vertically) in each photograph. For these reasons, KDEF material is particularly suitable for averaging validated expressions across individuals and for morphing between neutral and affect expressions. The prototypical reference portraits of affect expressive adult faces were created from the individual portraits of the KDEF picture-set using digital addition. 140 (68 male, 72 female) pictures were selected from a group of 490 pictures (group A, frontal) based on the highest validity (highest hit rate and intensity rating) according to the study of Goeleven and colleagues [34]. Each affect prototypical reference portrait was created using a suitable software (Abrosoft Fantamorph Deluxe 5) and consists of real faces of adults (KDEF; 7–13 portraits per average picture) and children (created as part of this study and the pilot study; 3–15 portraits per average picture) which have been digitally layered. The digital layering was made in the course of an averaging process using approx. 200 topical landmarks for every individual face. By means of these markers located i.g. on eyes, nose, mouth, eyebrows and ears, the single individual portraits of one specific affect cluster were layered and digitally averaged in a standardized procedure. Each real portrait was equally included in the final reference portrait. Due to this procedure the individual characteristics of the real faces disappeared and the specific basic affect was purified as a prototypical pattern. The obtained reference portraits were then further edited (*Adobe Photoshop Pro CS 6)* by making only face and hairline visible (Fig 2).

The original individual portraits of the real children are protected by law and are therefore not available, whereas the deindividualized affect prototypical averages of each basic affect exist in a male respective female version for scientific purposes available from the first author. The original individual portraits of the real adults are available via KDEF [16] and the derived affect prototypical averages are also available from the first author.

Additionally, the PSYCAFE portrait set and the affect expressive portraits based on the KDEF were used to create affect expressive video sequences fading from a neutral to a maximum expression for each basic affect of male and female adults and children by using Abrosoft Fantamorph Deluxe 5. These video sequences (3000 ms) showed a naturalistic and dynamic facial affect enrichment (2000 ms) and the subsequent static presentation of the apex of every basic affect (1000 ms).

### 2.4 Validation of the affect prototypical deindividualized portraits of children and adults

In a next step, the affect expressive deindividualized portraits of children and adults were validated by a healthy sample. Therefore, the digitally created portraits of children and adults were presented for assessment to a different group of healthy participants (N = 44) by using the presentation software PsychoPy [42]. Participants were recruited via social media as well as announcements for the study on the Internet. All participants gave their written informed consent to participate in the study. Portraits were presented on a 22-inch LCD-display with resolution of 1920x1080 (60 Hz). Participants were seated one meter in front of the screen. The rating-design was constructed analogously to the assessment of the original portraits of the real individuals. One of the digitally created reference portraits was presented once, and the participants were told to indicate which of the six basic affects they perceive while looking at the facial expression of the images. The specific affect could be selected using the keys 1–6. The sequence of visualisation of the selectable affects remained identical (happiness, fear, disgust, anger, surprise and sadness). Multiple responses were also possible. On the following screen page, participants were supposed to assess the intensity of the perceived facial expression. Entry could be made by pressing the keys 1–5 leading to possible values of 0 (no perceivable affect, therefore no perceived intensity) to 5 (very strong intensity of the perceived affect). Additionally, participants should rate the facial expressions regarding authenticity and likeability.

The descriptive specifications of the sample included mean values and frequency values of personal and psychometric data. The sample of raters assessing the digitally created affect prototypical reference portraits consisted of 23 (52%) women and 21 (48%) men. Mean age was *M* = 25.7 years (*SD* = 5.9) with a range from 18 to 44 years. 80% of participants were younger than 30 years. All participants had a university degree (45%) or high school graduation/Abitur (55%) as highest graduation. Other educational degrees were not represented in the current sample. Thirty-eight students (86%), three employees (7%), two self-employed individuals (5%) and one unemployed person (2%) took part in the test. The most common degree courses of the students were psychology (n = 9, 24%) and economics (n = 4, 11%).

As particularly high values in the TAS-20 (sum ≥ 61) and in the Hospital Anxiety and Depression Scale (HADS-D [43]; scale sum in one of the two scales ≥ 8) were defined as exclusion criteria, none of the participants showed high values in these scales. The mean TAS-20 sum score was *M* = 36.9 (*SD* = 7.8), the mean HADS depression score was *M* = 1.9 (*SD* = 1.9) and the mean HADS anxiety score was *M* = 3.0 (*SD* = 2.2). In order to validate the reference portraits, means of intensity values of the six basic affects were determined. Based on these values the index of difficulty (see method section) was calculated.

Subsequently, the hit rate, i.e. the ratio of participants who assigned the portrait to the intended affect, was calculated. Since a multiple selection of the inquired affects was possible, values of intensity were considered for the calculation of the hit rate. A result was counted as a hit if the highest value of intensity of a picture corresponded to its intended affect. A hit was not assessed if the highest intensity rating was not linked to the intended affect, or if the highest intensity rating of two affects had the same value.

Two-factorial repeated-measures analyses of variance (ANOVA) were conducted with *age* (child vs. adult) and *affect* (fear, disgust, happiness, sadness, surprise and anger) as factors and *hit rate*, *intensity*, *authenticity*, *and likeability* as the dependent variable.

As a further objective validation step beyond a mere observer rating, the stimulus material was imported in iMotions Biometric Research Platform 8.1 and analysed using Affectiva’s facial expression analysis software AFFDEX, an emotion recognition software [44, 45]. Because of the fact, that AFFDEX is not able to analyse single frames [46], only the affect expressive video sequences of the affects happiness and anger were analysed, because those two facial affects are widely used as stimuli in many studies. The results for the other basic affects will be published separately. AFFDEX is using a 30-second window to estimate the baseline appearance of each facial action [47]. In order to provide a sufficient baseline experience, the affect expressive videos of PSYCAFE were complemented with 30 seconds of displaying the neutral expression before the morphing sequence. Afterwards, probability values, which represent the likelihood that the video sequence expresses the expected affect, were computed for each affect expressive video sequence. These probability values were descriptively compared to the facial mimicry response of healthy adults to the video sequences, which was examined in a pre-study [37]. In this preliminary study, the electromyographically measured mimic activity related to the corrugator supercilii and zygomaticus muscles was investigated in response to those affect prototypical video clips of adults and children based on PSYCAFE and the deindividualized portraits of KDEF. A discrete mimic reaction was measured regarding each displayed affect (anger, disgust, happiness, sadness, surprise, anger), which can be interpreted as an additional criterion of validity of the stimulus material.

The entire study was approved by the responsible ethics commission of the Medical Faculty of the Heinrich-Heine-University (ID number 4998).

## 3. Results

### 3.1 Validation of the affect expressive portraits of children

The 197 participants of the online survey rated the 140 individual portraits of real children with regard to the intensity of the perceived affect. Using the calculated ratings of intensity, an index of difficulty was generated. Twelve of the 140 individual portraits were excluded from further analysis due to a negative index (S1 Table).

An empirical, hierarchical-agglomerative cluster analysis was performed on the remaining 128 pictures. The biggest difference of distance, i. e. the differences between the distance of the prior and the new cluster fusion, could be found in cluster solutions of 23, 18, 13, 10, 7 and 6. In order to obtain a differentiated assignment of the individual portraits to the respective clusters and to detect even small differences between the single clusters, the 23-cluster solution was chosen for further analysis.

Furthermore, it was investigated which of these clusters included images that were clearly attributable to a certain affect. After applying the cut-off criterion (see method section), 11 clusters (altogether consisting of 24 portraits) of the 23 clusters were excluded. Hence, 104 of the original 140 portraits were considered as valid. All 23 clusters could be grouped into seven resulting clusters (six basic affects and one neutral expression) by means of the highest and second highest intensity (for the respective affect). In a final step, this seven-cluster-solution was replicated and confirmed perfectly and in detail by a hierarchical cluster-analysis. The confirmation of the seven clusters and the assignment of the pictures to the clusters showed that this statistical classification was in accordance with the prior classification regarding the rates of intensity. Table 1 presents the final distribution of the 104 individual portraits to the seven clusters (6 basic affects and a neutral affect) including their intensity ratings and the index of difficulty.

**Table 1:** Rating of intensity of each basic affect and index of difficulty (range 0 to 5; difference between the highest affect rating and the added four other affect ratings) of the 104 individual affect expressive portraits of children and their distribution to the final 7 cluster.

ID	Clus-ter	f/m	intended affect	fea	SD_F_	dis	SD_D_	hap	SD_H_	sad	SD_S_	sur	SD_S_	ang	SD_A_	DI
**B05**	1	m	fear	2.42	1.85	0.98	1.58	0.00	0.00	0.13	0.70	0.58	1.28	0.14	0.78	0.59
**B08**	1	m	fear	2.83	1.56	0.43	1.21	0.00	0.00	0.03	0.30	0.42	1.12	0.03	0.30	1.92
**B09**	1	m	fear	3.06	2.11	0.05	0.36	0.03	0.30	0.00	0.00	1.50	2.03	0.17	0.79	1.31
**B12**	1	f	fear	2.98	1.83	0.19	0.76	0.03	0.31	0.33	1.07	0.48	1.20	0.00	0.00	1.95
**B19**	1	f	fear	2.34	1.69	0.89	1.45	0.00	0.00	0.00	0.00	0.74	1.44	0.06	0.44	0.65
**B21**	1	f	fear	2.36	1.78	0.63	1.35	0.00	0.00	0.00	0.00	0.90	1.56	0.00	0.00	0.83
**B22**	1	f	fear	3.59	1.79	0.25	0.87	0.19	0.78	0.00	0.00	0.85	1.71	0.08	0.56	2.22
**B23**	2	m	disgust	0.28	1.00	3.28	1.63	0.00	0.00	0.00	0.00	0.00	0.00	0.41	1.11	2.59
**B25**	2	m	disgust	0.00	0.00	2.49	1.51	0.58	1.27	0.03	0.31	0.04	0.41	0.16	0.66	1.68
**B28**	2	m	disgust	0.10	0.59	2.57	1.50	1.05	1.73	0.12	0.65	0.06	0.42	0.07	0.49	1.17
**B29**	2	m	disgust	0.03	0.30	2.82	1.54	0.56	1.18	0.00	0.00	0.15	0.67	0.03	0.30	2.05
**B30**	2	f	disgust	0.00	0.00	3.20	1.42	0.00	0.00	0.00	0.00	0.00	0.00	0.28	0.95	2.92
**B31**	2	f	disgust	0.01	0.10	2.69	1.33	0.28	0.88	0.03	0.31	0.01	0.10	0.04	0.41	2.32
**B32**	2	f	disgust	0.00	0.00	3.67	1.03	0.00	0.00	0.00	0.00	0.14	0.56	0.11	0.56	3.42
**B33**	2	f	disgust	0.07	0.51	3.20	1.45	0.00	0.00	0.04	0.41	0.08	0.48	0.22	0.83	2.79
**B35**	2	f	disgust	0.01	0.10	3.27	1.43	0.07	0.51	0.00	0.00	0.03	0.31	0.32	1.02	2.84
**B40**	2	f	disgust	0.00	0.00	2.63	1.65	0.37	1.06	0.00	0.00	0.21	0.79	0.19	0.70	1.86
**B41**	3	m	happiness	0.00	0.00	0.00	0.00	3.73	0.87	0.00	0.00	0.00	0.00	0.00	0.00	3.73
**B42**	3	m	happiness	0.00	0.00	0.00	0.00	4.39	0.73	0.00	0.00	0.02	0.21	0.00	0.00	4.37
**B43**	3	m	happiness	0.00	0.00	0.00	0.00	3.83	0.90	0.00	0.00	0.00	0.00	0.00	0.00	3.38
**B44**	3	m	happiness	0.00	0.00	0.00	0.00	4.16	0.62	0.00	0.00	0.00	0.00	0.00	0.00	4.16
**B45**	3	m	happiness	0.00	0.00	0.00	0.00	3.12	1.03	0.00	0.00	0.06	0.42	0.00	0.00	3.06
**B46**	3	m	happiness	0.00	0.00	0.00	0.00	3.12	1.26	0.00	0.00	0.00	0.00	0.00	0.00	3.12
**B47**	3	m	happiness	0.02	0.20	0.00	0.00	3.62	0.78	0.00	0.00	0.02	0.20	0.00	0.00	3.58
**B48**	3	m	happiness	0.05	0.36	0.00	0.00	3.25	0.93	0.01	0.10	0.09	0.53	0.00	0.00	3.10
**B49**	3	f	happiness	0.00	0.00	0.00	0.00	4.21	0.78	0.04	0.41	0.14	0.63	0.00	0.00	4.03
**B50**	3	f	happiness	0.00	0.00	0.00	0.00	4.11	0.69	0.00	0.00	0.00	0.00	0.00	0.00	4.11
**B51**	3	f	happiness	0.00	0.00	0.00	0.00	4.11	0.79	0.04	0.41	0.01	0.10	0.00	0.00	4.06
**B52**	3	f	happiness	0.00	0.00	0.00	0.00	3.78	0.95	0.00	0.00	0.00	0.00	0.00	0.00	3.78
**B53**	3	f	happiness	0.00	0.00	0.00	0.00	3.80	0.70	0.00	0.00	0.00	0.00	0.00	0.00	3.80
**B54**	3	f	happiness	0.03	0.22	0.00	0.00	3.92	0.81	0.00	0.00	0.00	0.00	0.00	0.00	3.89
**B55**	3	f	happiness	0.00	0.00	0.00	0.00	4.13	0.74	0.00	0.00	0.05	0.50	0.00	0.00	4.08
**B57**	3	f	happiness	0.00	0.00	0.02	0.20	4.03	0.83	0.00	0.00	0.11	0.55	0.00	0.00	3.90
**B58**	4	m	neutral	0.00	0.00	0.00	0.00	0.00	0.00	0.39	0.91	0.00	0.00	0.05	0.37	0.34
**B59**	4	m	neutral	0.00	0.00	0.02	0.21	0.04	0.25	0.03	0.23	0.00	0.00	0.00	0.00	-0.01
**B60**	4	m	neutral	0.07	0.42	0.01	0.10	0.00	0.00	0.01	0.10	0.00	0.00	0.01	0.10	0.04
**B61**	4	m	neutral	0.16	0.69	0.03	0.31	0.00	0.00	0.21	0.74	0.02	0.21	0.06	0.43	-0.06
**B62**	4	m	neutral	0.00	0.00	0.00	0.00	0.19	0.70	0.07	0.36	0.00	0.00	0.00	0.00	0.12
**B63**	4	m	neutral	0.00	0.00	0.00	0.00	0.09	0.39	0.02	0.14	0.02	0.21	0.00	0.00	0.05
**B64**	4	m	neutral	0.00	0.00	0.00	0.00	0.06	0.37	0.00	0.00	0.00	0.00	0.00	0.00	0.06
**B65**	4	m	neutral	0.00	0.00	0.00	0.00	0.19	0.59	0.05	0.36	0.02	0.20	0.00	0.00	0.12
**B66**	4	m	neutral	0.00	0.00	0.00	0.00	0.09	0.43	0.39	1.07	0.00	0.00	0.00	0.00	0.30
**B67**	4	m	neutral	0.03	0.22	0.00	0.00	0.02	0.20	0.46	1.06	0.00	0.00	0.06	0.42	0.35
**B68**	4	m	neutral	0.00	0.00	0.00	0.00	0.07	0.29	0.03	0.30	0.00	0.00	0.00	0.00	0.04
**B69**	4	f	neutral	0.03	0.31	0.00	0.00	0.03	0.23	0.18	0.67	0.00	0.00	0.00	0.00	0.12
**B70**	4	f	neutral	0.04	0.32	0.00	0.00	0.00	0.00	0.41	0.96	0.00	0.00	0.00	0.00	0.37
**B71**	4	f	neutral	0.03	0.31	0.00	0.00	0.15	0.60	0.12	0.56	0.00	0.00	0.00	0.00	0.00
**B72**	4	f	neutral	0.00	0.00	0.00	0.00	0.14	0.52	0.00	0.00	0.00	0.00	0.00	0.00	0.14
**B73**	4	f	neutral	0.00	0.00	0.00	0.00	0.01	0.10	0.20	0.69	0.00	0.00	0.00	0.00	0.19
**B74**	4	f	neutral	0.27	0.90	0.00	0.00	0.01	0.10	0.48	1.10	0.00	0.00	0.00	0.00	0.20
**B75**	4	f	neutral	0.01	0.10	0.00	0.00	0.27	0.82	0.03	0.30	0.03	0.30	0.00	0.00	0.20
**B76**	4	f	neutral	0.03	0.30	0.02	0.20	0.00	0.00	0.24	0.88	0.00	0.00	0.08	0.48	0.11
**B77**	4	f	neutral	0.00	0.00	0.00	0.00	0.22	0.61	0.00	0.00	0.00	0.00	0.00	0.00	0.22
**B78**	4	f	neutral	0.02	0.20	0.00	0.00	0.00	0.00	0.11	0.51	0.00	0.00	0.04	0.24	0.05
**B79**	5	m	sadness	0.22	0.95	0.00	0.00	0.00	0.00	2.99	1.31	0.00	0.00	0.15	0.62	2.62
**B80**	5	m	sadness	0.19	0.79	0.00	0.00	0.06	0.43	2.51	1.33	0.00	0.00	0.23	0.81	2.03
**B81**	5	m	sadness	0.00	0.00	0.00	0.00	0.00	0.00	2.80	1.48	0.00	0.00	0.64	1.34	2.16
**B82**	5	m	sadness	0.13	0.62	0.05	0.37	0.00	0.00	2.76	1.32	0.02	0.14	0.02	0.21	2.54
**B83**	5	m	sadness	0.03	0.31	0.01	0.10	0.00	0.00	3.08	1.26	0.00	0.00	0.19	0.66	2.85
**B84**	5	m	sadness	0.00	0.00	0.00	0.00	0.00	0.00	2.20	1.49	0.02	0.20	0.46	1.07	1.72
**B85**	5	m	sadness	0.05	0.36	0.00	0.00	0.00	0.00	2.73	1.31	0.00	0.00	0.22	0.82	2.46
**B86**	5	m	sadness	0.03	0.30	0.00	0.00	0.00	0.00	3.68	0.91	0.00	0.00	0.00	0.00	3.65
**B87**	5	m	sadness	0.02	0.20	0.02	0.20	0.00	0.00	3.12	1.25	0.00	0.00	0.05	0.26	3.03
**B88**	5	m	sadness	0.18	0.70	0.00	0.00	0.00	0.00	2.18	1.29	0.00	0.00	0.12	0.51	1.88
**B89**	5	f	sadness	0.05	0.37	0.01	0.10	0.00	0.00	2.60	1.42	0.00	0.00	0.33	0.86	2.21
**B90**	5	f	sadness	0.00	0.00	0.00	0.00	0.00	0.00	2.56	1.29	0.00	0.00	0.33	1.01	2.23
**B91**	5	f	sadness	0.00	0.00	0.00	0.00	0.00	0.00	2.43	1.45	0.00	0.00	0.04	0.41	2.39
**B92**	5	f	sadness	0.27	0.90	0.02	0.21	0.00	0.00	3.94	1.00	0.00	0.00	0.22	0.80	3.43
**B93**	5	f	sadness	0.00	0.00	0.05	0.34	0.00	0.00	2.69	1.28	0.00	0.00	0.04	0.32	2.60
**B94**	5	f	sadness	0.05	0.36	0.00	0.00	0.00	0.00	2.71	1.61	0.03	0.30	0.82	1.48	1.81
**B97**	5	f	sadness	0.33	0.96	0.29	0.92	0.00	0.00	2.14	1.59	0.01	0.10	0.18	0.59	1.33
**B98**	5	f	sadness	0.03	0.30	0.04	0.28	0.00	0.00	1.90	1.47	0.00	0.00	0.46	1.05	1.37
**B100**	6	m	surprise	0.00	0.00	0.00	0.00	0.48	1.15	0.00	0.00	3.41	1.20	0.00	0.00	2.93
**B101**	6	m	surprise	1.13	1.66	0.01	0.10	0.00	0.00	0.03	0.31	2.82	1.64	0.00	0.00	1.65
**B103**	6	m	surprise	0.06	0.43	0.00	0.00	0.62	1.38	0.00	0.00	3.73	1.28	0.00	0.00	3.05
**B104**	6	m	surprise	0.00	0.00	0.07	0.49	0.16	0.70	0.00	0.00	3.63	1.10	0.00	0.00	3.40
**B105**	6	m	surprise	0.59	1.32	0.02	0.20	0.05	0.36	0.00	0.00	2.94	1.60	0.01	0.10	2.27
**B106**	6	m	surprise	0.25	0.94	0.04	0.40	0.96	1.73	0.00	0.00	3.59	1.66	0.00	0.00	2.34
**B107**	6	m	surprise	0.56	1.37	0.03	0.30	0.11	0.54	0.00	0.00	4.02	1.38	0.00	0.00	3.32
**B108**	6	m	surprise	0.58	1.26	0.00	0.00	0.00	0.00	0.00	0.00	3.12	1.43	0.00	0.00	2.54
**B109**	6	m	surprise	1.28	1.92	0.11	0.66	0.00	0.00	0.00	0.00	3.58	1.85	0.00	0.00	2.19
**B110**	6	f	surprise	1.12	1.72	0.01	0.10	0.00	0.00	0.05	0.37	3.19	1.71	0.00	0.00	2.01
**B111**	6	f	surprise	0.94	1.62	0.00	0.00	0.04	0.29	0.00	0.00	3.38	1.61	0.00	0.00	2.40
**B112**	6	f	surprise	1.02	1.70	0.01	0.10	0.08	0.52	0.00	0.00	3.64	1.76	0.00	0.00	2.53
**B115**	6	f	surprise	0.62	1.42	0.02	0.21	0.00	0.00	0.02	0.21	3.35	1.37	0.00	0.00	2.69
**B116**	6	f	surprise	0.73	1.37	0.00	0.00	0.09	0.45	0.03	0.22	2.54	1.53	0.00	0.00	1.69
**B117**	6	f	surprise	0.62	1.31	0.00	0.00	0.01	0.10	0.00	0.00	2.81	1.43	0.00	0.00	2.18
**B118**	6	f	surprise	0.07	0.50	0.00	0.00	0.05	0.30	0.00	0.00	3.66	0.78	0.00	0.00	3.54
**B119**	6	f	surprise	0.21	0.84	0.04	0.40	0.30	1.07	0.00	0.00	4.35	1.13	0.00	0.00	3.80
**B120**	6	f	surprise	0.47	1.21	0.04	0.40	0.00	0.00	0.00	0.00	3.57	1.28	0.00	0.00	3.06
**B122**	7	m	anger	0.01	0.10	0.13	0.72	0.00	0.00	0.16	0.70	0.00	0.00	3.99	1.08	3.69
**B124**	7	m	anger	0.03	0.31	0.04	0.41	0.00	0.00	0.17	0.74	0.00	0.00	4.02	1.14	3.78
**B126**	7	m	anger	0.02	0.21	0.03	0.31	0.00	0.00	0.58	1.24	0.00	0.00	3.24	1.21	2.61
**B127**	7	m	anger	0.04	0.28	0.07	0.50	0.00	0.00	0.04	0.40	0.00	0.00	3.76	1.10	3.61
**B129**	7	m	anger	0.00	0.00	0.00	0.00	0.00	0.00	0.31	0.99	0.00	0.00	3.13	1.28	2.82
**B131**	7	f	anger	0.08	0.58	0.00	0.00	0.00	0.00	0.02	0.21	0.00	0.00	3.28	1.20	3.18
**B133**	7	f	anger	0.00	0.00	0.28	0.95	0.00	0.00	0.03	0.31	0.03	0.31	2.88	1.44	2.54
**B135**	7	f	anger	0.12	0.65	0.00	0.00	0.00	0.00	0.23	0.92	0.00	0.00	3.54	1.06	3.19
**B136**	7	f	anger	0.07	0.43	0.37	1.05	0.00	0.00	0.00	0.00	0.02	0.20	2.71	1.39	2.25
**B137**	7	f	anger	0.05	0.50	0.02	0.20	0.00	0.00	0.00	0.00	0.00	0.00	4.49	0.85	4.42
**B138**	7	f	anger	0.29	1.01	0.22	0.84	0.00	0.00	0.45	1.14	0.00	0.00	3.27	1.72	2.31
**B139**	7	f	anger	0.00	0.00	1.12	1.88	0.00	0.00	0.06	0.42	0.00	0.00	3.08	1.92	1.90
**B140**	7	f	Anger	0.00	0.00	0.35	0.99	0.05	0.36	0.00	0.00	0.00	0.00	2.36	1.46	1.96

ID = portrait number. SD = standard deviation. f = female, m = male, fea = fear, dis = disgust, hap = happiness, sad = sadness, sur = surprise, ang = anger, DI = index of difficulty.

In order to add already FACS-coded [27] real faces of children out of the pilot study [26] to the set, the cut-off criterion was also applied to the clusters of the pilot study. Three clusters (cluster 10, 18, 19) with a total of 13 images had to be excluded. A further image from this set was excluded due to the child’s unfavourable position of the head (B68).

### 3.2 Validation of the affect prototypical deindividualized portraits of children and adults

As the difficulty-index rated by the healthy subjects (N = 44; mentioned above) of all 24 deindividualized portraits was positive (Table 2), all images were assigned by high intensity to a certain affect. Hit rates and intensity were computed for each affect expressive portrait.

**Table 2:** Values of intensity, difficulty index (DI; range 0 to 5; difference between the highest affect rating and the added four other affect ratings) and hit rates (HR; in %) of the 24 assessed affect prototypical reference pictures.

age	sex	affect	N_IP_	fear (SD)	disgust (SD)	happiness (SD)	sadness (SD)	surprise (SD)	anger (SD)	DI	HR (SD)
**adult**	**male**	**fear**	12	**3.91 (**1.16)	0.07 (0.45)	0.00 (0.00)	0.27 (1.04)	1.23 (1.76)	0.00 (0.00)	2.34	81.82 (39.02)
		**disgust**	11	0.00 (0.00)	**4.11 (**0.72)	0.00 (0.00)	0.09 (0.42)	0.07 (0.45)	0.50 (1.23)	3.45	95.45 (21.07)
		**happiness**	10	0.00 (0.00)	0.00 (0.00)	**4.16 (**0.71)	0.00 (0.00)	0.00 (0.00)	0.00 (0.00)	4.16	100.00 (0.00)
		**sadness**	9	0.05 (0.30)	0.00 (0.00)	0.00 (0.00)	**3.93 (**1.09)	0.23 (1.09)	0.00 (0.00)	3.65	97.73 (15.08)
		**surprise**	7	0.30 (0.79)	0.00 (0.00)	0.05 (0.21)	0.00 (0.00)	**4.16 (**0.81)	0.00 (0.00)	3.81	100.00 (0.00)
		**anger**	9	0.11 (0.62)	0.16 (0.75)	0.00 (0.00)	0.14 (0.63)	0.00 (0.00)	**4.64 (**0.53)	4.23	97.73 (15.08)
	**female**	**fear**	8	**4.30 (**0.63)	0.18 (0.62)	0.00 (0.00)	0.77 (1.46)	0.07 (0.45)	0.00 (0.00)	3.28	90.91 (29.08)
		**disgust**	9	0.00 (0.00)	**4.16 (**1.31)	0.00 (0.00)	0.02 (0.15)	0.05 (0.30)	0.59 (1.34)	3.50	93.18 (25.50)
		**happiness**	10	0.00 (0.00)	0.00 (0.00)	**4.18 (**0.81)	0.00 (0.00)	0.07 (0.45)	0.00 (0.00)	4.11	100.00 (0.00)
		**sadness**	11	0.27 (0.73)	0.00 (0.00)	0.00 (0.00)	**3.93 (**0.76)	0.00 (0.00)	0.00 (0.00)	3.66	100.00 (0.00)
		**surprise**	13	0.00 (0.00)	0.00 (0.00)	1.25 (1.51)	0.00 (0.00)	**4.07 (**0.95)	0.00 (0.00)	2.82	93.18 (25.50)
		**anger**	11	0.00 (0.00)	0.16 (0.81)	0.00 (0.00)	0.00 (0.00)	0.00 (0.00)	**3.91 (**1.07)	3.75	95.45 (21.07)
**child**	**male**	**fear**	3	**4.05 (**1.57)	0.32 (1.05)	0.02 (0.15)	0.05 (0.30)	1.30 (1.94)	0.00 (0.00)	2.36	77.27 (42.39)
		**disgust**	9	0.00 (0.00)	**2.80 (**1.29)	0.27 (0.87)	0.00 (0.00)	0.05 (0.30)	0.73 (1.34)	1.75	77.27 (42.39)
		**happiness**	12	0.00 (0.00)	0.00 (0.00)	**3.82 (**1.17)	0.05 (0.30)	0.00 (0.00)	0.02 (0.15)	3.75	95.45 (21.07)
		**sadness**	12	0.11 (0.54)	0.05 (0.30)	0.00 (0.00)	**3.55 (**1.21)	0.00 (0.00)	0.16 (0.68)	3.23	93.18 (25.50)
		**surprise**	10	0.07 (0.45)	0.00 (0.00)	1.02 (1.52)	0.00 (0.00)	**4.70 (**0.51)	0.00 (0.00)	3.61	95.45 (21.07)
		**anger**	7	0.05 (0.30)	0.05 (0.30)	0.00 (0.00)	0.52 (1.21)	0.00 (0.00)	**3.84 (**0.75)	3.22	93.18 (25.50)
	**female**	**fear**	5	**3.50 (**1.36)	0.43 (1.07)	0.07 (0.33)	0.00 (0.00)	1.18 (1.73)	0.00 (0.00)	1.82	79.55 (40.80)
		**disgust**	10	0.00 (0.00)	**3.82 (**0.69)	0.07 (0.45)	0.00 (0.00)	0.00 (0.00)	0.27 (0.90)	3.48	95.45 (21.07)
		**happiness**	15	0.00 (0.00)	0.00 (0.00)	**4.07 (**0.79)	0.00 (0.00)	0.18 (0.87)	0.00 (0.00)	3.89	97.73 (15.08)
		**sadness**	10	0.25 (0.78)	0.02 (0.15)	0.00 (0.00)	**3.02 (**1.25)	0.00 (0.00)	0.16 (0.57)	2.59	93.18 (25.50)
		**surprise**	15	0.32 (0.88)	0.00 (0.00)	0.43 (1.11)	0.00 (0.00)	**4.39 (**0.69)	0.00 (0.00)	3.64	95.45 (21.07)
		**anger**	12	0.00 (0.00)	0.17 (0.63)	0.00 (0.00)	0.30 (0.95)	0.00 (0.00)	**3.36 (**1.10)	2.89	95.45 (21.07)

SD = standard deviation; N_IP =_ number of individual portraits that were used to create the affect prototypical reference pictures.

**Fig 3:**
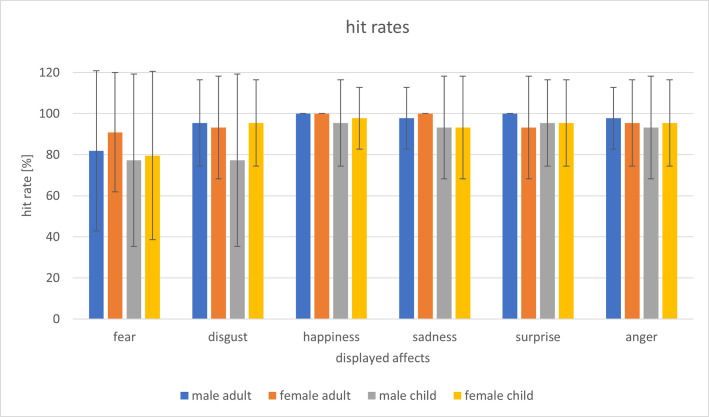
Hit rates of the affect prototypical reference pictures of the six basic affects of male and female adults and children. The error bars represent standard deviations. Since all participants recognized the portraits “happiness male / female adult”, “sadness female adult” and “surprise male adult” 100% correctly, the standard deviations were 0.

The mean hit rate was 93% (*SD* = 7.80). Hit rates of the single images ranged from 77% to 100%. Fig 3 provides an overview of the single hit rates for the presented basic affects of adults and children.

Using a two-factorial repeated-measures ANOVA with *age* (child vs. adult) and *affect* (fear, disgust, happiness, sadness, surprise, anger) as factors and *hit rate* as dependent variable, a main effect for *age*, *F*(1,43) = 10.75; *p* < .01; *η*^*2*^ = .200, as well as for *affect*, *F*(5,215) = 6.70; *p* < .01; *η*^*2*^ = 0.135, was revealed. There was no significant interaction between both factors, *F*(5,215) = 0.76; *p* = .540; *η*^*2*^ = .017. The images of adults (*M* = 95%; *SD* = 7.93) showed significantly higher hit rates than the images of children (*M* = 91%; *SD* = 10.20). Generally, anxious faces had a significantly lower hit rate (*M* = 82%; *SD* = 27.80) than facial expressions of happiness (*M* = 98%; *SD* = 6.37; *p* < .01), sadness (*M* = 96%, *SD* = 10.71; *p* = .045), surprise (*M* = 96%; *SD* = 10.71; *p* = .033) and anger (*M* = 95%; *SD* = 9.75; *p* = .048). Further pairwise comparisons showed no significant differences of hit rates.

**Fig 4:**
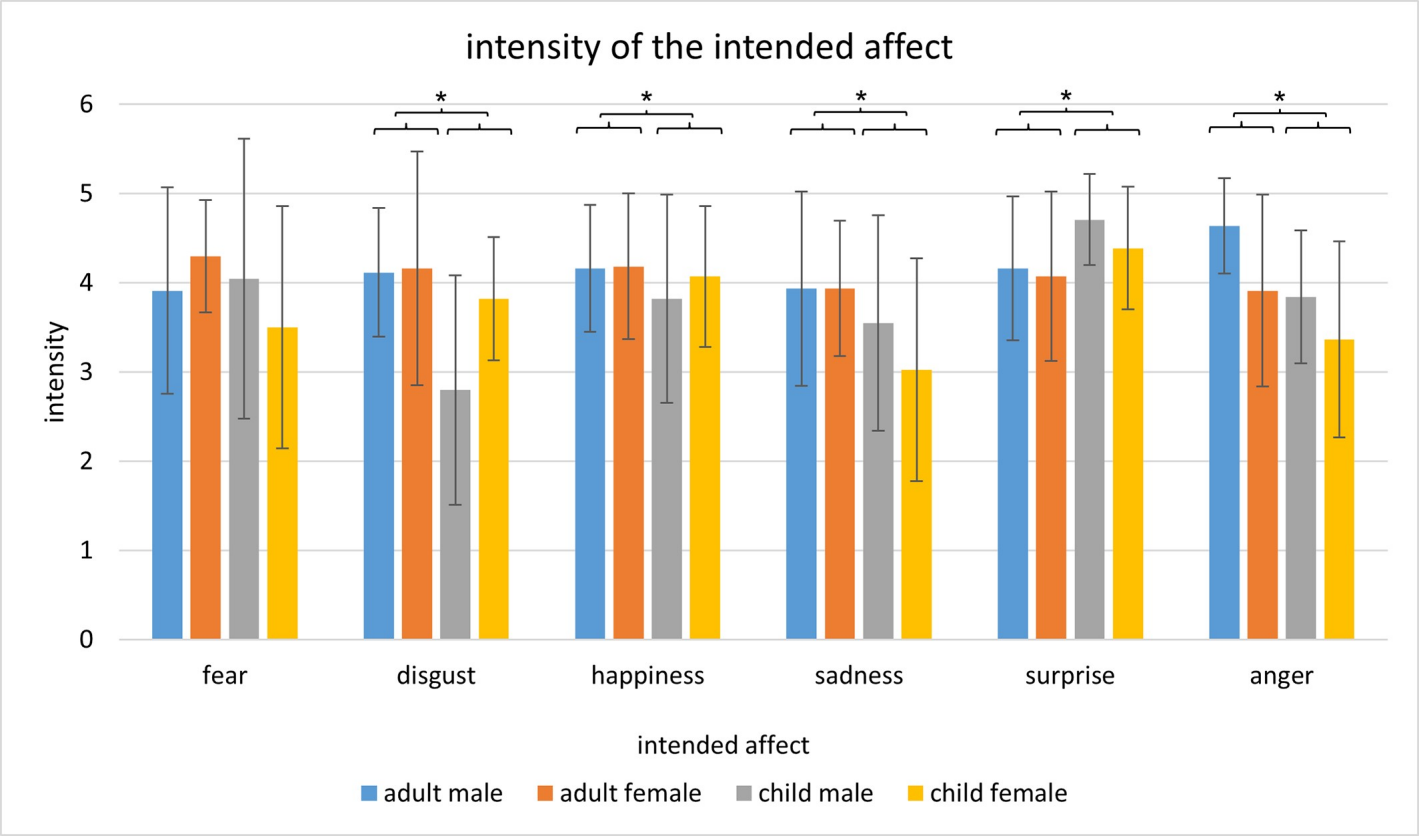
Mean intensity of the intended affect for adults’ and children’s affect expressive faces. The error bars represent the standard deviation. Significant post hoc tests of adults’ and children’s faces (not seperated per sex) are presented as following * < .05.

Moreover, the mean estimations of intensity of the intended affects indicated the validity of the respective image. As shown in Fig 4, the mean estimations of intensity of the intended affect of children’s portraits ranged between *M* = 2.80 (*SD* = 1.29) and *M* = 4.70 (*SD* = 0.51). Those of adults ranged between *M* = 3.91 (*SD* = 1.07) and *M* = 4.64 (*SD* = 0.53). The intensity was estimated as strong (4) to very strong (5) with regard to adults’ images and medium (3) to very strong (5) regarding children’s images.

For closer examination, a two-factorial repeated-measures ANOVA with *age* (child vs. adult) and *affect* (fear, disgust, happiness, sadness, surprise and anger) as factors and *intensity* as dependent variable was computed. There was a main effect for *age*, *F*(1,43) = 33.91; *p* < .01; *η*^*2*^ = .441, for *affect*, *F*(5,215) = 7.45; *p* < .01; *η*^*2*^ = .148, and a significant interaction for *age* x *affect*, *F*(5,215) = 11.27; *p* < .01; *η*^*2*^ = .208. All affect expressive portraits of children (*M* = 3.74; *SD* = 0.47) were assessed as less intense than the portraits of adults (*M* = 4.12; *SD* = 0.38).

Regarding pairwise comparisons of the variable *affect*, the facial expression of surprise was rated as significant more intense (*M* = 4.33, *SD* = 0.47) compared to disgust (*M* = 3.72, *SD* = 0.70; *p* < .01), sadness (*M* = 3.61, *SD* = 0.82; *p* < .01) and anger (*M* = 3.94; *SD* = 0.45; *p* < .01). Intensity of happiness (*M* = 4.06; *SD* = 0.71) was estimated significantly higher than sadness (*M* = 3.61; *SD* = 0.82; *p* < .01). Further pairwise comparisons obtained no significant results.

Pairwise comparisons of the interaction *age* x *affect* revealed significant differences in the assessment of intensity between portraits of adults and children with regard to the following affects: disgust (*M*_adults_ = 4.14; *SD* = 0.83; *M*_children_ = 3.31; *SD* = 0.82; *p* < .01), happiness (*M*_adults_ = 4.17; *SD* = 0.70; *M*_children_ = 3.94; *SD* = 0.88; *p* = .04), sadness (*M*_adults_ = 3.93; *SD* = 0.80; *M*_children_ = 3.28; *SD* = 1.08; *p* < .01), surprise (*M*_adults_ = 4.11; *SD* = 0.66; *M*_children_ = 4.55; *SD* = 0.52; *p* < .01) and anger (*M*_adults_ = 4.27; *SD* = 0.60; *M*_children_ = 3.60; *SD* = 0.70; *p* < .01).

Apart from hit rates and intensity, the authenticity of the affect expressive faces was assessed. A two-factorial repeated-measures ANOVA for *age* and *affect* as factors and *authenticity* as dependent variable indicates a significant main effect for *age*, *F*(1,43) = 4.73; *p* = .035; *η*^*2*^ = .099, *affect*, *F*(5,215) = 34.15; *p* < .01; *η*^*2*^ = .443, and a significant interaction for *age* x *affect*, *F*(5,215) = 7.53; *p* < .01; *η*^*2*^ = .149.

Images of children (*M* = 3.88; *SD* = 0.53) were experienced as significantly more authentic than images of adults (*M* = 3.75; *SD* = 0.52).

**Fig 5:**
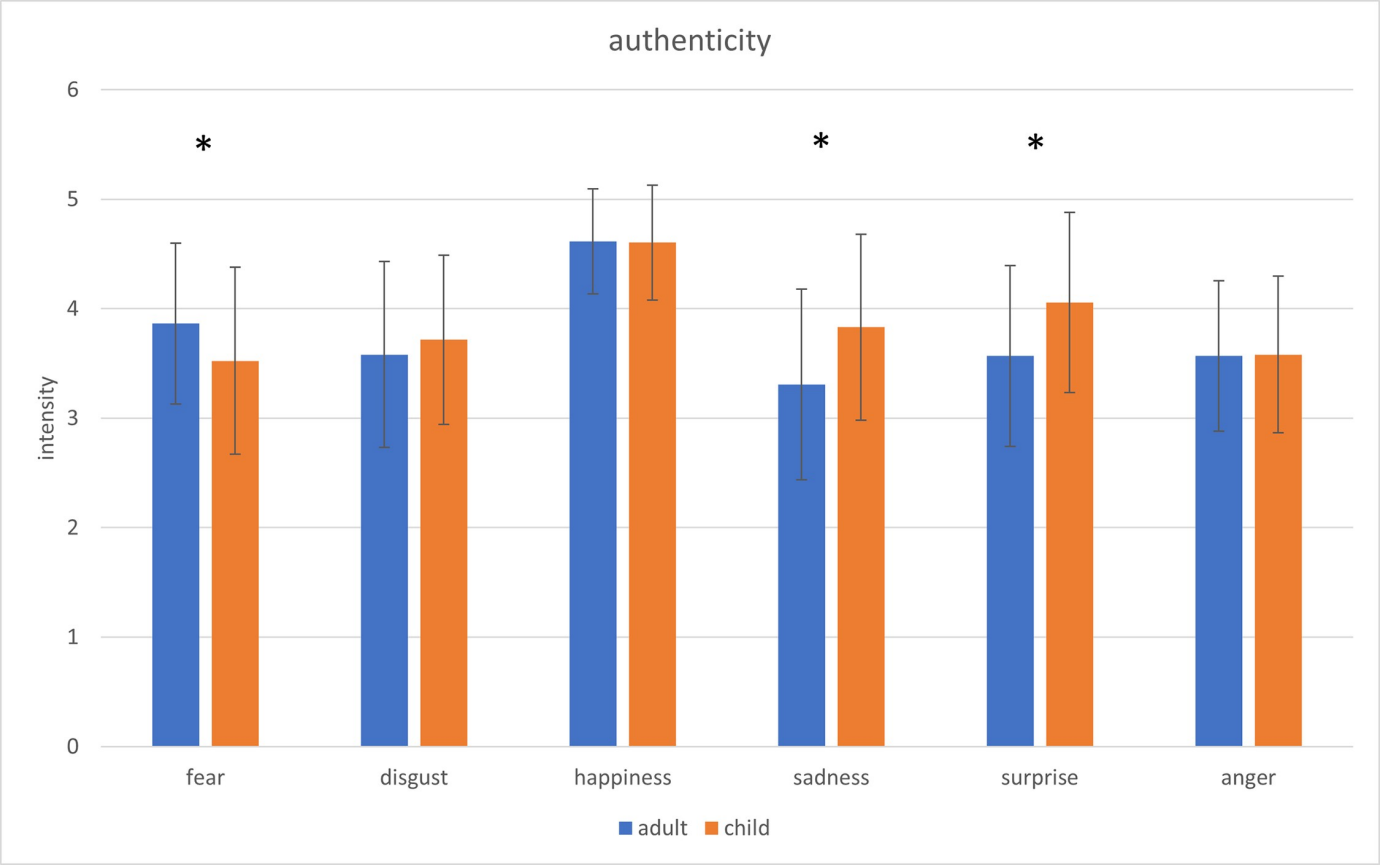
Comparison of the ratings of intensity regarding authenticity of the presented adults’ and children’s affect expressive faces. The error bars represent the standard deviation. * = p < .05.

Fig 5 depicts the ratings of intensity of the presented adults’ and children’s affect expressive faces with regard to authenticity.

A similar ANOVA with likeability as dependent variable revealed a significant main effect for *age*, *F* (1,43) = 15.07; *p* < .01; *η*^*2*^ = .259; for *affect*, *F* (5,215) = 123.94; *p* < .01; *η*^*2*^ = .742 and a significant interaction for *age* x *affect*, *F* (5,215) = 5.74; *p* < .01; *η*^*2*^ = .118.

**Fig 6:**
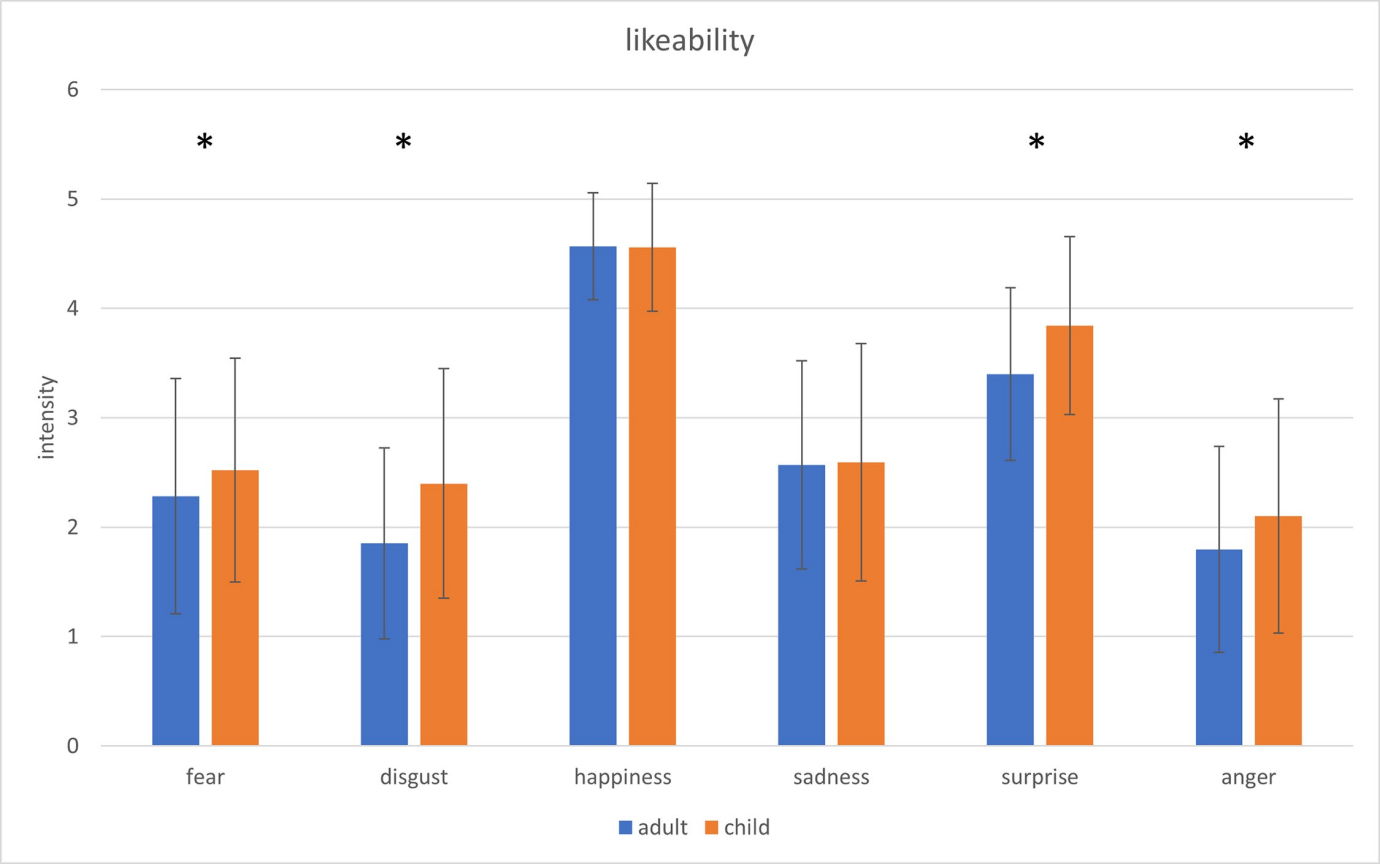
Comparison of the ratings of intensity regarding likeability of the presented adults’ and children’s affect expressive faces. The error bars represent the standard deviation. * = p < .05.

The affect prototypical portraits of children (*M* = 3.00; *SD* = 0.70) were assessed as significantly more likeable than images of adults (*M* = 2.74; *SD* = 0.64). The interaction *age* x *affect* was also analysed using pairwise comparisons showing significantly higher assessments of likeability concerning children’s images of fear, disgust, anger and surprise compared to images of adults (Fig 6).

**Fig 7:**
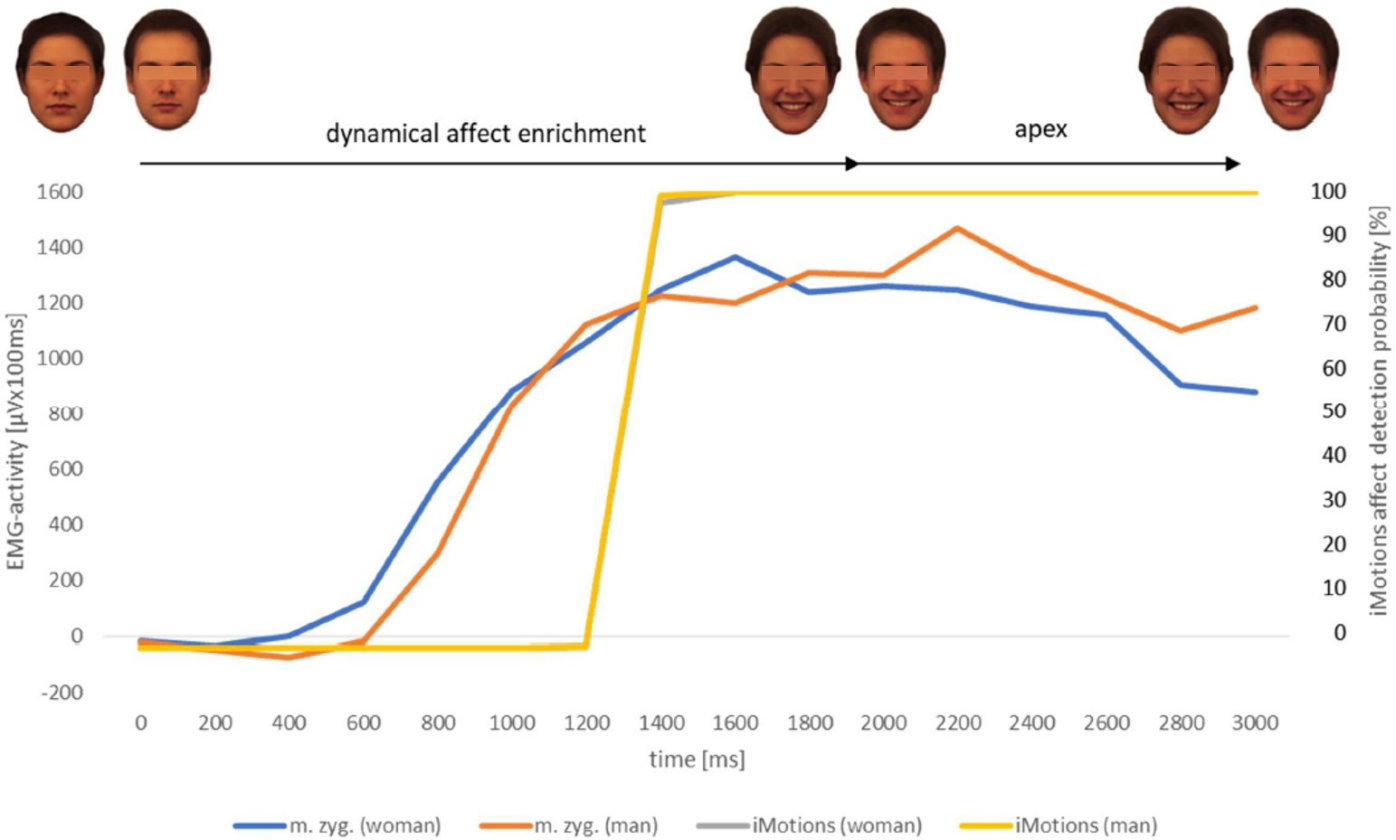
EMG-activity (N = 43) of m. zygomaticus [μVx100ms] of healthy observers in response to the presented videoclips displaying the facial expression of happiness naturalistic evolving from a neutral face of the female respective male reference portrait; software based (AFFDEX/iMotions) affect detection probability values [%] of the same presented stimuli; measurement interval for both procedures 3000ms. m.zyg. (woman) = EMG-activity of musculus zygomaticus in response to the adult female face video. m.zyg. (man) = EMG-activity of musculus zygomaticus in response to the adult male face video. iMotions (woman) = software based affect detection probability [%] in response to the adult female face video. iMotions (man) = software based affect detection probability [%] in response to the adult male face video. The yellow (man) and grey (woman) lines are superposed within this graph because of a nearly similar time course.

**Fig 8:**
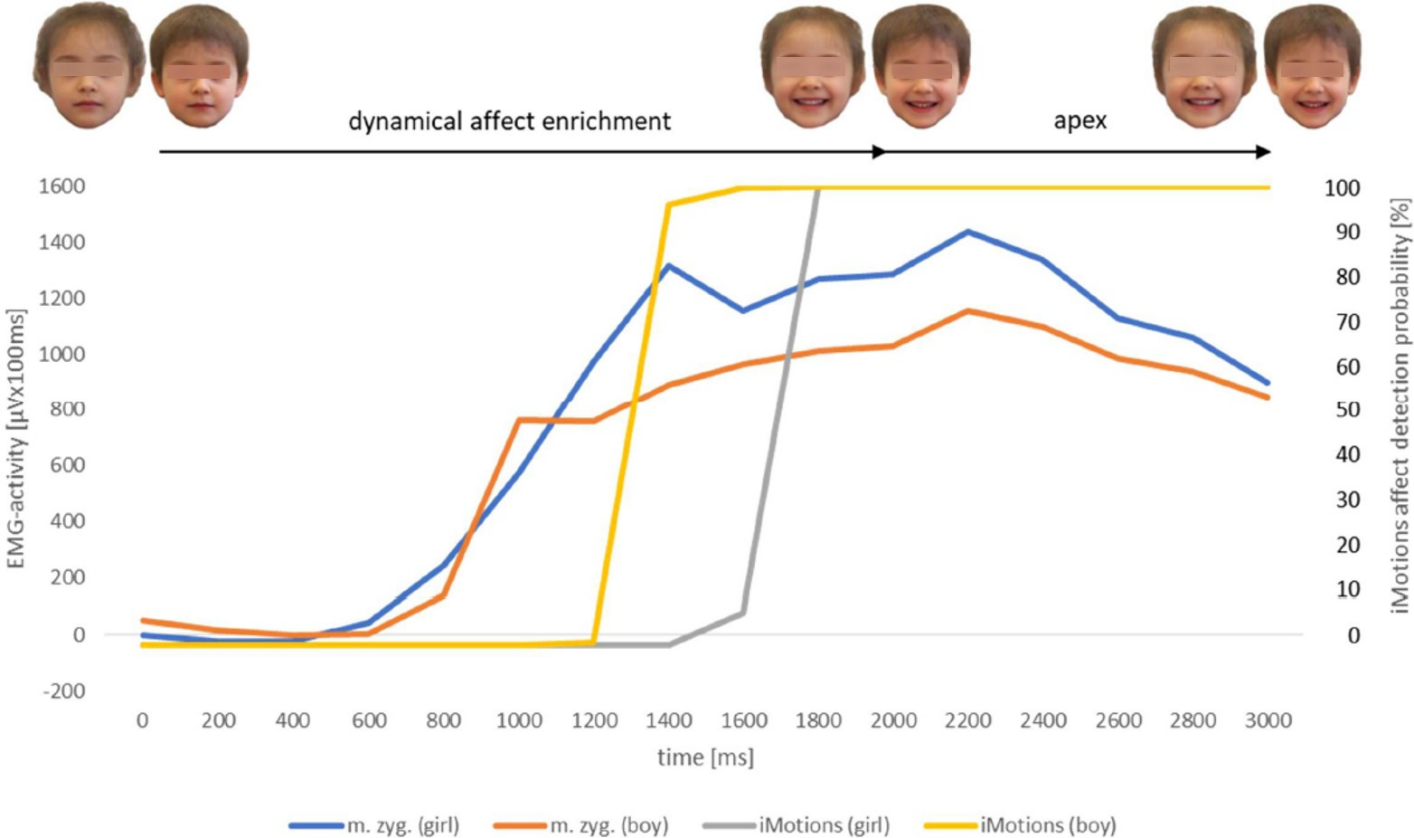
EMG-activity (N = 43) of m. zygomaticus [μVx100ms] of healthy observers in response to the presented videoclips displaying the facial expression of happiness naturalistic evolving from a neutral face of the girl’s respective boy’s reference portrait; software based (AFFDEX/iMotions) affect detection probability values [%] of the same presented stimuli; measurement interval for both procedures 3000ms. m.zyg. (girl) = EMG-activity of musculus zygomaticus in response to the girl’s face video. m.zyg. (boy) = EMG-activity of musculus zygomaticus in response to the boy’s face video. iMotions (girl) = software based affect detection probability [%] in response to the girl’s face video. iMotions (boy) = software based affect detection probability [%] in response to the boy’s face video. The yellow (boy) and grey (girl) lines are superposed in part within this graph because of a partly similar time course.

**Fig 9:**
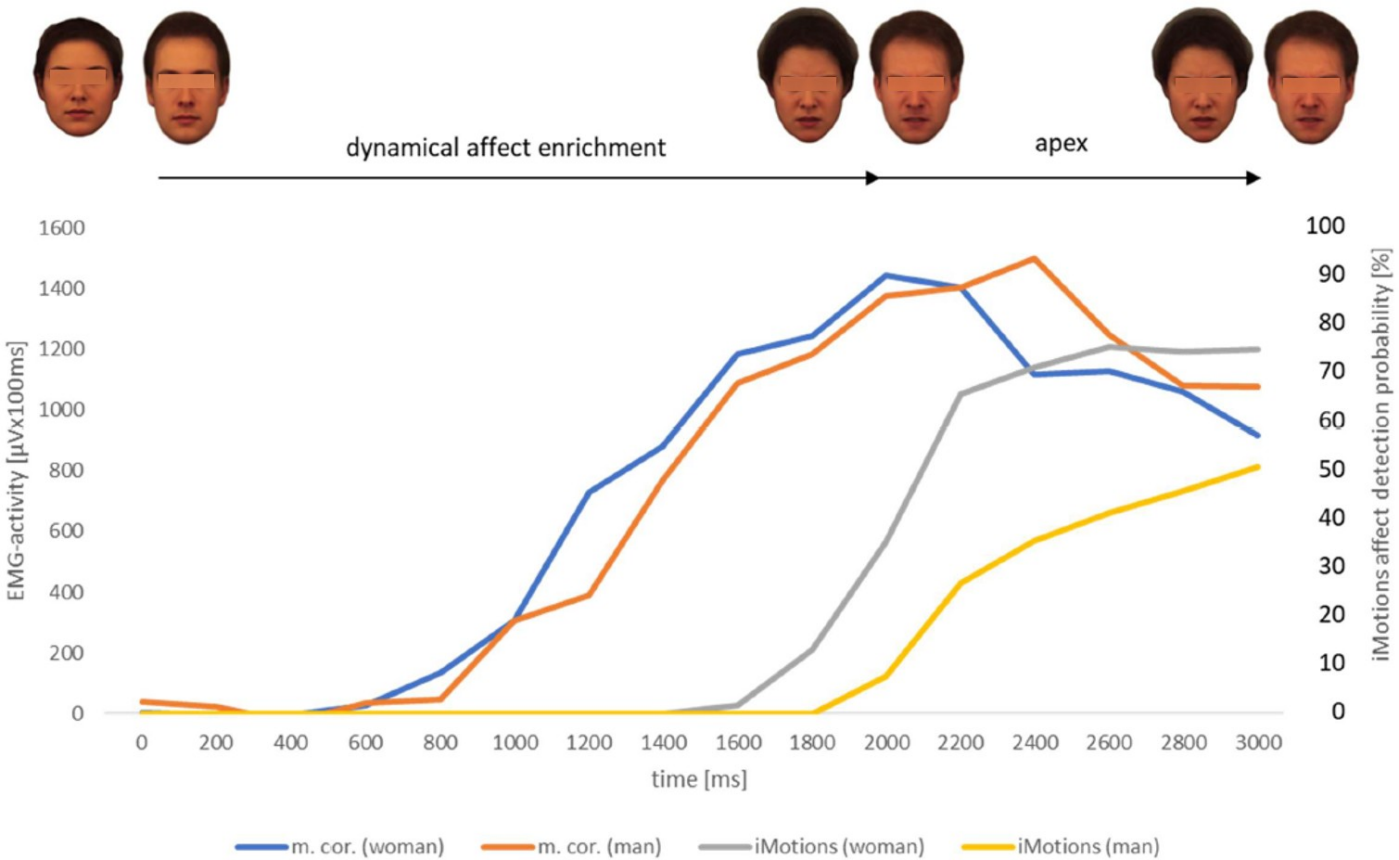
EMG-activity (N = 43) of m. corrugator [μVx100ms] of healthy observers in response to the presented videoclips displaying the facial expression of anger naturalistic evolving from a neutral face of the female respective male reference portrait; software based (AFFDEX/iMotions) affect detection probability values [%] of the same presented stimuli; measurement interval for both procedures 3000ms. m.cor. (woman) = EMG-activity of musculus corrugator in response to the adult female face video. m.cor. (man) = EMG-activity of musculus corrugator in response to the adult male face video. iMotions (woman) = software based affect detection probability [%] in response to the adult female face video. iMotions (man) = software based affect detection probability [%] in response to the adult male face video.

**Fig 10:**
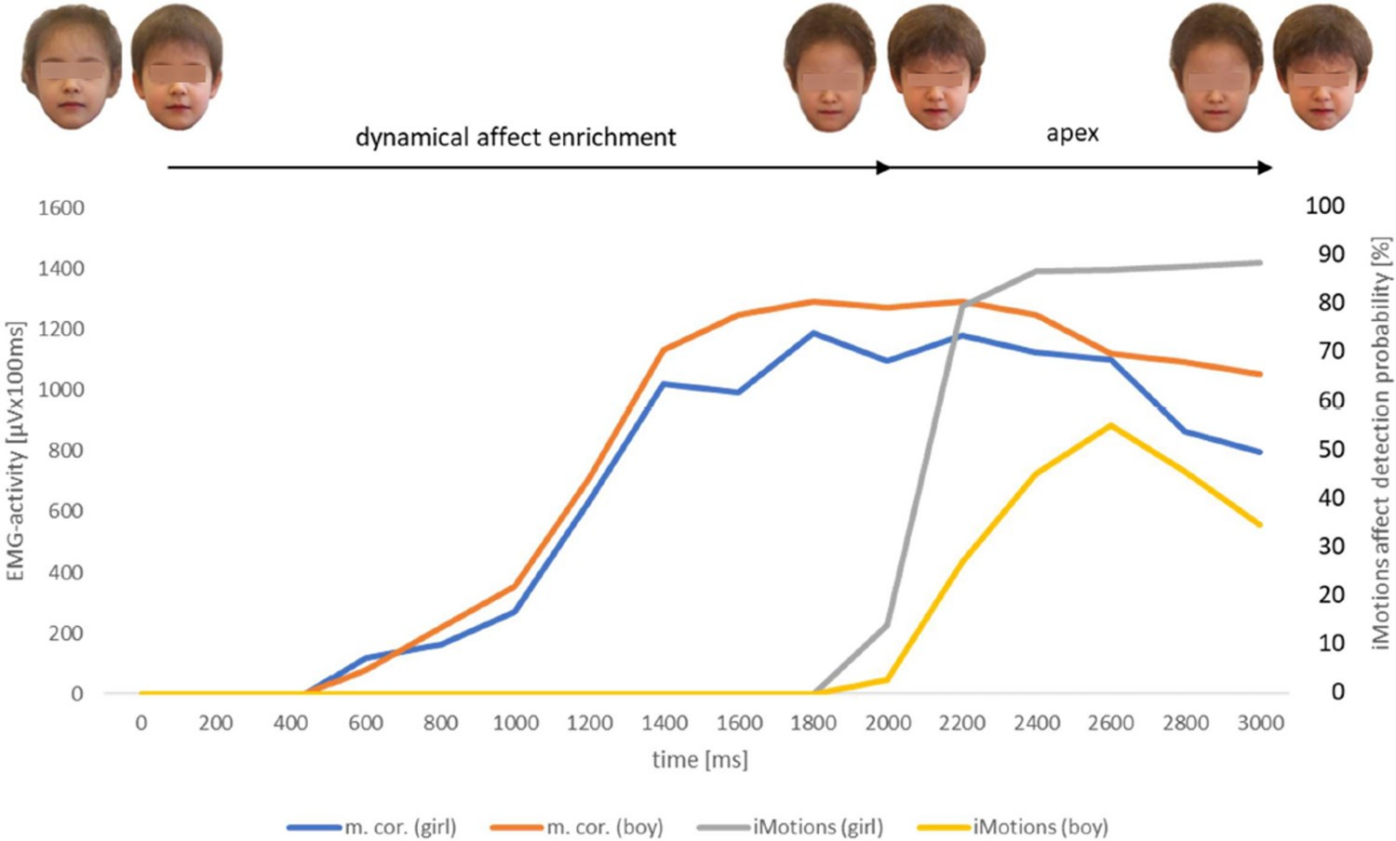
EMG-activity (N = 43) of m. corrugator [μVx100ms] of healthy observers in response to the presented videoclips displaying the facial expression of anger naturalistic evolving from a neutral face of the girl’s respective boy’s reference portrait; software based (AFFDEX/iMotions) affect detection probability values [%] of the same presented stimuli; measurement interval for both procedures 3000ms. m.cor. (girl) = EMG-activity of musculus corrugator in response to the girl’s face video. m.cor. (boy) = EMG-activity of musculus corrugator in response to the boy’s face video. iMotions (girl) = software based affect detection probability [%] in response to the girl’s face video. iMotions (boy) = software based affect detection probability [%] in response to the boy’s face video.

The analysis of the affect expressive video sequences by the facial affect recognition software AFFDEX can be taken as a further indication of validity regarding the affects anger and happiness (Figs 7–10). The probability values of the affect happiness were up to 100% in all groups (man, woman, boy and girl). Those rates of the affect anger were up to 50.83% (man), 74.89% (woman), 55.24% (boy) and 88.64% (girl).

## 4. Discussion

In this report, we present a newly developed and validated picture-set of children’s affective facial expressions. By using 12 reference portraits of children aged 4 to 6 years that were digitally layered and subsequently deindividualized, novel emotional stimulus material was created which was previously unavailable to the field of affective research. Under methodological aspects, this study confirms that theatre workshops are appropriate to create a picture-set of affective faces of pre-school children [26].

A large sample of 197 participants assigned 104 of the 140 selected individual portraits of real children to one of the six basic affects. In combination with 45 already validated pictures (FACS-coding and rating of affect intensity) deriving from a pilot study [26], the starting material for the PSYCAFE picture-set included 104 additional individual portraits resulting in an entire picture-set of N = 149 (82 female and 67 male). These 104 additional portraits were not validated by FACS, but within an extensive rating process as mentioned above. These individual portraits of children were finally used for the creation of digitally layered affect prototypical reference portraits.

The deindividualized portraits prevent children who took part in the workshops from being identified in future studies, as it is the case in other portrait sets [22, 23]. Besides, it is expected that individual features of faces are attenuated and the common characteristics of the specific affect become more explicit.

Both the digitally layered affect prototypical portraits of the children’s faces of the PSYCAFE and the adults’ reference portraits of the KDEF picture-set achieved high hit rates from 77% up to 100% and therefore can be considered as valid. Compared to the faces of adults, the basic affects expressed by preschool children were identified with lowered intensities and hit rates. Nevertheless, the hit rates of the portraits of children and their assessed intensities were high and close to these of the adults. The hit rates of the Picture-Set of Young Children’s Affective Facial Expressions obtained similar or even higher values than the hit rates of other picture-sets [19, 32, 34]. Portraits, which display the basic affect of happiness, showed the highest hit rate whereas pictures presenting the affect of fear received the lowest hit rates of both children and adults. The pictures of the affect disgust also got lower hit-rates than other affects, a pattern that appeared in the validation of other picture-sets as well [19, 32, 34].

The hit rates of other picture-sets as FACES [19] or the NimStim picture-set [18] were calculated via single-choice questions, whereas this study used a multiple-choice format. Therefore, it was necessary to adapt the multiple response model to the single response model in order to compare hit rates between the picture-sets. Assuming that in the case of a single choice format the selected option represents the highest intensity, hit rates were calculated using the highest intensity rating. This method was also used in a study of Hess and Blairy [48]. On this basis, the hit rates of the PSYCAFE can be compared with hit rates of other sets.

The PSYCAFE provides valid stimulus material for research on the perception and the effect of pre-school children’s affective facial expression. The differential validity of the children’s affect expressive portraits was shown meanwhile in psychophysiological EMG- studies of the facial mimicry in response to this stimulus material [37]. In this study, an affect specific response of the facial mimicry was demonstrated dependent from the presented affect expressive children’s portraits. PSYCAFE enables experimental studies on perception in the field of attachment behaviour and developmental psychology, particularly with regard to differences in perception and processing of basic affects shown by affective faces of both children and adults. It also enables to compare detection performance of clinical sub-groups concerning mimic expressions of children and adults and to measure the impact of the baby schema on affect detection.

Limitations of PSYCAFE relate specifically to difficulties in obtaining affect-expressive individual portraits of young children with respect to specific unhedonic affects such as fear. Due to practical reasons and their impulsive play behaviour, it is not easy to attain authentic mimic affect expressions of preschool children under standardized conditions. During the appropriate contact with the children in a free play situation, it was not possible to generate the same number of individual face expressions for every basic affect. This is true particularly for the induction of fear, which is restricted also for ethical reasons. Compared to other facial affect expressions (N = 5–15) the number of real individuals is diminished for this basic affect (N = 3). Nevertheless, the identification hit rate of the digitally deindividualized facial expression of fear is not diminished for the children compared to adults (Fig 3), which were digitally fused from 7 up to 13 real individual portraits. However, at least for the basic affects happiness and anger, which are mainly used within perception studies, the number of the individual source portraits is sufficient. Additionally, affect expressive portraits of children were assessed as more authentic compared to those of adults. This suggests that the described procedure is useful for the generation of valid stimulus material. In fact, differential response effects (facial EMG) of the PSYCAFE stimulus set could be demonstrated within experimental psychophysiological perception studies [37]. The results of the analysis by AFFDEX can be taken as a further validation criterion. In contrast to the facial EMG, which only refers to specific muscle activity, AFFDEX includes the activity of several facial parts and therefore provides a differentiated valuation of the stimuli. Nevertheless, the facial activity (fEMG) of the participants in reaction to the affect expressive video sequences began to increase after 500-1000ms [37], whereas AFFDEX identified the correct affect of the presented stimuli at a later point. These differences between the facial reaction and the AFFDEX probability scores with reference to the stimuli or even to a videoclip of the participants facial reaction are an interesting point to consider in future studies. Other studies have also shown that these software-based analyses of affect expressive faces are helpful for the categorization of different expressions, but currently seem to be limited, especially in the differentiation of blended expressions [49]. The video sequences with the basic affects happiness and anger, which we generated from digitally processed reference portraits that have been created from real individual children’s faces, were recognized by the AFFDEX software in the same way or even slightly better than the stimulus material of adult faces created from the KDEF. This could be taken as an indication that the design and validation process of PSYCAFE is comparable in outcome to that of the KDEF, although a relatively smaller number of individual source images were available.

The fact that the children’s pictures were rated as more likeable as the respective adults’ pictures based on KDEF indicates the importance of the baby schema. The study of Glocker and colleagues [4] demonstrates similar results. They showed that faces with distinct features of the baby schema were perceived as more positive and involved a stronger motivation to care for the child. Further studies confirmed that children’s faces appear to be more likeable and attractive than adults’ faces [50] and revealed a tendency of adults to protect and care for individuals with distinct features of the baby schema [51]. The present finding that children’s faces are rated as significantly more likeable as the corresponding adults’ faces, affirms the important role of parental affect regulation and sensitivity in the perception of children’s faces and provides a basis for future studies on the relation of the baby schema and affect expression. The effect of the baby schema on facial mimicry has already been demonstrated by using PSYCAFE [37, 52]. Müller et al. [37] found a diminished EMG-activity of the m. corrugator supercilii of participants watching anhedonic facial affect expressions of children compared to adults.

As PSYCAFE includes the single basic affects as well as a male and female neutral portrait, faces with a gradual expression located between the neutral and the maximum facial affect expression can also be created. Faces showing a less pronounced basic affect are important for performance-studies working with clinical groups who are impaired in the perception and processing of facial affect expressions (e.g. due to depression, alexithymia, autism spectrum disorders). First clinical studies demonstrated a significantly diminished facial mimicry of depressive patients in response to the PSYCAFE children’s portraits compared to controls [53].

In addition to gradually stepped affect expression of static single pictures, PSYCAFE includes dynamic naturalistic video sequences starting with a neutral face expression and ending with the apex of a certain affect [37, 53]. Such dynamic stimulus material is more suitable for affect induction than static single pictures [54]. It can be used for psychophysiological studies on affect processing similar to those of Isomura & Nakano [55], Hess & Blairy [48] or Rymarczyk et al. [54].

Factors considered to have a direct effect on mimicry were subject of numerous studies. These studies also investigated differential effects in terms of static and dynamic specification of visual stimuli [14], the relationship between facial mimicry and empathy [56, 57] and the impact of subthreshold priming [15]. However, there are only few studies in the fields of perception and processing of affect expressive children’s faces [58, 59]. Such picture-sets are of great value for research in the fields of affect processing, attachment, childhood development and affect related responsive behaviour. PSYCAFE provides the first picture-set of affect-prototypical reference portraits, which were created from individual, affect expressive portraits of children aged 4 to 6 years using a digital morphing procedure.

It can facilitate further studies on affect perception and processing without violating personal rights of children who mostly are not able to consider the consequences of wide spreading their own portraits.

## 5. Conclusion

In summary, PSYCAFE is a validated picture-set of deindividualized facial portraits of boys and girls aged 4 to 6 years representing the six basic affects as well as a neutral face. The digitally deindividualized portraits of the PSYCAFE picture-set were created from 149 individual pictures of real children in play. In an analogous manner, digitally processed deindividualized, prototypical average facial affect portraits of male and female adults were created from the individual affect expressive faces of the KDEF, which served as reference. The present study provides identification hit rates and values for intensity, authenticity and likeability of the specific affect displayed. The identification probability with regard to the basic affects happiness and anger by the facial affect recognition software AFFDEX supports the validity of PSYCAFE. PSYCAFE is the first picture-set including digitally created prototypical affect expressive pictures of the age group of pre-schoolers and is available as stimulus material for further studies by contacting the first author. Statistical Data and the deindividualized stimulus material can be found at https://doi.org/10.6084/m9.figshare.15070167.v1 and https://doi.org/10.6084/m9.figshare.15073845.v1.

## Supporting information

S1 Table Rating of intensity of each basic affect and index of difficulty (range 0 to 5; difference between the highest affect rating and the added four other affect ratings) of the 140 individual affect expressive portraits of children taking part in the online-rating. Gray = Portraits were excluded due to negative index of difficulty. Yellow: highest rating of intensity. SD = standard deviation. f = female. m = male.
(DOCX)
