# Supplementary material for: Creation and validation of the Picture-Set of Young Children’s Affective Facial Expressions (PSYCAFE)
Source: PLoS One. 2021 Dec 7;16(12):e0260871. doi: 10.1371/journal.pone.0260871 (PMC8651117; doi:10.1371/journal.pone.0260871)
Supplement: S1 Table — Gray = Portraits were excluded due to negative index of difficulty. Yellow: highest rating of intensity. SD = standard deviation. f = female. m = male. (DOCX) [file pone.0260871.s001.docx]

S**1 Table. Rating of intensity of each basic affect and index of difficulty (range 0 to 5; difference between the highest affect rating and the added four other affect ratings) of the 140 individual affect expressive portraits of children taking part in the online-rating.** Gray = Portraits were excluded due to negative index of difficulty. Yellow: highest rating of intensity. SD = standard deviation. f = female. m = male.

| picture | f/m | intended affect | fear | SD_F_ | disgust | SD_D_ | happiness | SD_H_ | sadness | SD_S_ | surprise | SD_S_ | anger | SD_A_ | index of difficulty |
| --- | --- | --- | --- | --- | --- | --- | --- | --- | --- | --- | --- | --- | --- | --- | --- |
| B01 | m | fear | 2.24 | 2.10 | 0.06 | 0.52 | 0.63 | 1.45 | 0.03 | 0.31 | 1.88 | 2.12 | 0.00 | 1.00 | -0.36 |
| B02 | m | fear | 1.22 | 1.61 | 0.40 | 0.98 | 0.00 | 0.00 | 0.00 | 0.00 | 1.24 | 1.70 | 0.94 | 1.54 | -1.32 |
| B03 | m | fear | 1.44 | 1.73 | 0.39 | 1.12 | 0.00 | 0.00 | 0.00 | 0.00 | 1.86 | 1.83 | 0.00 | 0.00 | 0.03 |
| B04 | m | fear | 1.89 | 1.97 | 1.47 | 1.84 | 0.00 | 0.00 | 0.86 | 1.60 | 0.07 | 0.44 | 0.08 | 0.58 | -0.59 |
| B05 | m | fear | 2.42 | 1.85 | 0.98 | 1.58 | 0.00 | 0.00 | 0.13 | 0.70 | 0.58 | 1.28 | 0.14 | 0.78 | 0.59 |
| B06 | m | fear | 1.54 | 1.51 | 0.96 | 1.46 | 0.19 | 0.66 | 0.30 | 0.31 | 0.24 | 0.82 | 0.00 | 0.00 | -0.15 |
| B07 | m | fear | 2.06 | 1.88 | 1.25 | 1.84 | 0.00 | 0.00 | 0.00 | 0.00 | 0.65 | 1.43 | 0.25 | 0.95 | -0.09 |
| B08 | m | fear | 2.83 | 1.56 | 0.43 | 1.21 | 0.00 | 0.00 | 0.03 | 0.30 | 0.42 | 1.12 | 0.03 | 0.30 | 1.92 |
| B09 | m | fear | 3.06 | 2.11 | 0.05 | 0.36 | 0.03 | 0.30 | 0.00 | 0.00 | 1.50 | 2.03 | 0.17 | 0.79 | 1.31 |
| B10 | m | fear | 2.14 | 2.19 | 0.10 | 0.50 | 0.44 | 1.29 | 0.00 | 0.00 | 1.05 | 1.87 | 0.81 | 1.58 | -0.26 |
| B11 | m | fear | 1.39 | 1.74 | 1.60 | 1.87 | 0.39 | 1.06 | 0.04 | 0.40 | 0.50 | 1.29 | 0.00 | 0.00 | -0.72 |
| B12 | f | fear | 2.98 | 1.83 | 0.19 | 0.76 | 0.03 | 0.31 | 0.33 | 1.07 | 0.48 | 1.20 | 0.00 | 0.00 | 1.95 |
| B13 | f | fear | 0.57 | 1.16 | 1.25 | 1.53 | 0.05 | 0.37 | 0.00 | 0.00 | 1.15 | 1.53 | 0.00 | 0.00 | -0.52 |
| B14 | f | fear | 2.24 | 2.10 | 0.06 | 0.43 | 0.00 | 0.00 | 0.00 | 0.00 | 2.62 | 1.97 | 0.00 | 0.00 | 0.32 |
| B15 | f | fear | 1.58 | 2.09 | 0.04 | 0.41 | 1.25 | 1.95 | 0.00 | 0.00 | 2.44 | 2.07 | 0.00 | 0.00 | -0.43 |
| B16 | f | fear | 1.98 | 1.79 | 0.20 | 0.74 | 0.00 | 0.00 | 0.06 | 0.38 | 1.74 | 1.78 | 0.00 | 0.00 | -0.02 |
| B17 | f | fear | 1.75 | 2.14 | 0.17 | 0.85 | 1.42 | 2.11 | 0.04 | 0.40 | 1.64 | 2.11 | 0.00 | 0.00 | -1.52 |
| B18 | f | fear | 2.58 | 2.02 | 1.66 | 2.03 | 0.00 | 0.00 | 0.08 | 0.58 | 0.26 | 0.96 | 0.07 | 0.50 | 0.51 |
| B19 | f | fear | 2.34 | 1.69 | 0.89 | 1.45 | 0.00 | 0.00 | 0.00 | 0.00 | 0.74 | 1.44 | 0.06 | 0.44 | 0.65 |
| B20 | f | fear | 1.53 | 1.76 | 0.31 | 0.97 | 0.00 | 0.00 | 0.52 | 1.23 | 1.70 | 1.81 | 0.00 | 0.00 | -1.00 |
| B21 | f | fear | 2.36 | 1.78 | 0.63 | 1.35 | 0.00 | 0.00 | 0.00 | 0.00 | 0.90 | 1.56 | 0.00 | 0.00 | 0.83 |
| B22 | f | fear | 3.59 | 1.79 | 0.25 | 0.87 | 0.19 | 0.78 | 0.00 | 0.00 | 0.85 | 1.71 | 0.08 | 0.56 | 2.22 |
| B23 | m | disgust | 0.28 | 1.00 | 3.28 | 1.63 | 0.00 | 0.00 | 0.00 | 0.00 | 0.00 | 0.00 | 0.41 | 1.11 | 2.59 |
| B24 | m | disgust | 0.00 | 0.00 | 1.52 | 1.49 | 0.00 | 0.00 | 0.00 | 0.00 | 0.14 | 0.63 | 1.02 | 1.47 | 0.36 |
| B25 | m | disgust | 0.00 | 0.00 | 2.49 | 1.51 | 0.58 | 1.27 | 0.03 | 0.31 | 0.04 | 0.41 | 0.16 | 0.66 | 1.68 |
| B26 | m | disgust | 0.04 | 0.31 | 1.40 | 1.47 | 0.98 | 1.34 | 0.01 | 0.10 | 0.08 | 0.46 | 0.08 | 0.34 | 0.21 |
| B27 | m | disgust | 0.25 | 0.83 | 1.99 | 1.84 | 0.00 | 0.00 | 0.14 | 0.64 | 0.04 | 0.40 | 1.07 | 1.54 | 0.49 |
| B28 | m | disgust | 0.10 | 0.59 | 2.57 | 1.50 | 1.05 | 1.73 | 0.12 | 0.65 | 0.06 | 0.42 | 0.07 | 0.49 | 1.17 |
| B29 | m | disgust | 0.03 | 0.30 | 2.82 | 1.54 | 0.56 | 1.18 | 0.00 | 0.00 | 0.15 | 0.67 | 0.03 | 0.30 | 2.05 |
| B30 | f | disgust | 0.00 | 0.00 | 3.20 | 1.42 | 0.00 | 0.00 | 0.00 | 0.00 | 0.00 | 0.00 | 0.28 | 0.95 | 2.92 |
| B31 | f | disgust | 0.01 | 0.10 | 2.69 | 1.33 | 0.28 | 0.88 | 0.03 | 0.31 | 0.01 | 0.10 | 0.04 | 0.41 | 2.32 |
| B32 | f | disgust | 0.00 | 0.00 | 3.67 | 1.03 | 0.00 | 0.00 | 0.00 | 0.00 | 0.14 | 0.56 | 0.11 | 0.56 | 3.42 |
| B33 | f | disgust | 0.07 | 0.51 | 3.20 | 1.45 | 0.00 | 0.00 | 0.04 | 0.41 | 0.08 | 0.48 | 0.22 | 0.83 | 2.79 |
| B34 | f | disgust | 0.04 | 0.41 | 2.36 | 1.84 | 0.00 | 0.00 | 0.00 | 0.00 | 0.00 | 0.00 | 1.39 | 1.74 | 0.93 |
| B35 | f | disgust | 0.01 | 0.10 | 3.27 | 1.43 | 0.07 | 0.51 | 0.00 | 0.00 | 0.03 | 0.31 | 0.32 | 1.02 | 2.84 |
| B36 | f | disgust | 0.11 | 0.56 | 2.20 | 1.70 | 0.00 | 0.00 | 0.02 | 0.20 | 0.00 | 0.00 | 1.07 | 1.51 | 1.00 |
| B37 | f | disgust | 0.10 | 0.61 | 2.63 | 1.82 | 0.00 | 0.00 | 0.10 | 0.52 | 0.03 | 0.30 | 1.04 | 1.64 | 1.36 |
| B38 | f | disgust | 0.07 | 0.49 | 3.01 | 1.69 | 0.00 | 0.00 | 0.23 | 0.80 | 0.06 | 0.42 | 0.34 | 1.00 | 2.31 |
| B39 | f | disgust | 0.00 | 0.00 | 2.31 | 2.00 | 0.00 | 0.00 | 1.16 | 1.76 | 0.03 | 0.30 | 0.40 | 1.07 | 0.72 |
| B40 | f | disgust | 0.00 | 0.00 | 2.63 | 1.65 | 0.37 | 1.06 | 0.00 | 0.00 | 0.21 | 0.79 | 0.19 | 0.70 | 1.86 |
| B41 | m | happiness | 0.00 | 0.00 | 0.00 | 0.00 | 3.73 | 0.87 | 0.00 | 0.00 | 0.00 | 0.00 | 0.00 | 0.00 | 3.73 |
| B42 | m | happiness | 0.00 | 0.00 | 0.00 | 0.00 | 4.39 | 0.73 | 0.00 | 0.00 | 0.02 | 0.21 | 0.00 | 0.00 | 4.37 |
| B43 | m | happiness | 0.00 | 0.00 | 0.00 | 0.00 | 3.83 | 0.90 | 0.00 | 0.00 | 0.00 | 0.00 | 0.00 | 0.00 | 3.38 |
| B44 | m | happiness | 0.00 | 0.00 | 0.00 | 0.00 | 4.16 | 0.62 | 0.00 | 0.00 | 0.00 | 0.00 | 0.00 | 0.00 | 4.16 |
| B45 | m | happiness | 0.00 | 0.00 | 0.00 | 0.00 | 3.12 | 1.03 | 0.00 | 0.00 | 0.06 | 0.42 | 0.00 | 0.00 | 3.06 |
| B46 | m | happiness | 0.00 | 0.00 | 0.00 | 0.00 | 3.12 | 1.26 | 0.00 | 0.00 | 0.00 | 0.00 | 0.00 | 0.00 | 3.12 |
| B47 | m | happiness | 0.02 | 0.20 | 0.00 | 0.00 | 3.62 | 0.78 | 0.00 | 0.00 | 0.02 | 0.20 | 0.00 | 0.00 | 3.58 |
| B48 | m | happiness | 0.05 | 0.36 | 0.00 | 0.00 | 3.25 | 0.93 | 0.01 | 0.10 | 0.09 | 0.53 | 0.00 | 0.00 | 3.10 |
| B49 | f | happiness | 0.00 | 0.00 | 0.00 | 0.00 | 4.21 | 0.78 | 0.04 | 0.41 | 0.14 | 0.63 | 0.00 | 0.00 | 4.03 |
| B50 | f | happiness | 0.00 | 0.00 | 0.00 | 0.00 | 4.11 | 0.69 | 0.00 | 0.00 | 0.00 | 0.00 | 0.00 | 0.00 | 4.11 |
| B51 | f | happiness | 0.00 | 0.00 | 0.00 | 0.00 | 4.11 | 0.79 | 0.04 | 0.41 | 0.01 | 0.10 | 0.00 | 0.00 | 4.06 |
| B52 | f | happiness | 0.00 | 0.00 | 0.00 | 0.00 | 3.78 | 0.95 | 0.00 | 0.00 | 0.00 | 0.00 | 0.00 | 0.00 | 3.78 |
| B53 | f | happiness | 0.00 | 0.00 | 0.00 | 0.00 | 3.80 | 0.70 | 0.00 | 0.00 | 0.00 | 0.00 | 0.00 | 0.00 | 3.80 |
| B54 | f | happiness | 0.03 | 0.22 | 0.00 | 0.00 | 3.92 | 0.81 | 0.00 | 0.00 | 0.00 | 0.00 | 0.00 | 0.00 | 3.89 |
| B55 | f | happiness | 0.00 | 0.00 | 0.00 | 0.00 | 4.13 | 0.74 | 0.00 | 0.00 | 0.05 | 0.50 | 0.00 | 0.00 | 4.08 |
| B56 | f | happiness | 0.00 | 0.00 | 0.00 | 0.00 | 1.73 | 1.77 | 0.00 | 0.00 | 0.00 | 0.00 | 0.04 | 0.40 | 1.69 |
| B57 | f | happiness | 0.00 | 0.00 | 0.02 | 0.20 | 4.03 | 0.83 | 0.00 | 0.00 | 0.11 | 0.55 | 0.00 | 0.00 | 3.90 |
| B58 | m | neutral | 0.00 | 0.00 | 0.00 | 0.00 | 0.00 | 0.00 | 0.39 | 0.91 | 0.00 | 0.00 | 0.05 | 0.37 | 0.34 |
| B59 | m | neutral | 0.00 | 0.00 | 0.02 | 0.21 | 0.04 | 0.25 | 0.03 | 0.23 | 0.00 | 0.00 | 0.00 | 0.00 | -0.01 |
| B60 | m | neutral | 0.07 | 0.42 | 0.01 | 0.10 | 0.00 | 0.00 | 0.01 | 0.10 | 0.00 | 0.00 | 0.01 | 0.10 | 0.04 |
| B61 | m | neutral | 0.16 | 0.69 | 0.03 | 0.31 | 0.00 | 0.00 | 0.21 | 0.74 | 0.02 | 0.21 | 0.06 | 0.43 | -0.06 |
| B62 | m | neutral | 0.00 | 0.00 | 0.00 | 0.00 | 0.19 | 0.70 | 0.07 | 0.36 | 0.00 | 0.00 | 0.00 | 0.00 | 0.12 |
| B63 | m | neutral | 0.00 | 0.00 | 0.00 | 0.00 | 0.09 | 0.39 | 0.02 | 0.14 | 0.02 | 0.21 | 0.00 | 0.00 | 0.05 |
| B64 | m | neutral | 0.00 | 0.00 | 0.00 | 0.00 | 0.06 | 0.37 | 0.00 | 0.00 | 0.00 | 0.00 | 0.00 | 0.00 | 0.06 |
| B65 | m | neutral | 0.00 | 0.00 | 0.00 | 0.00 | 0.19 | 0.59 | 0.05 | 0.36 | 0.02 | 0.20 | 0.00 | 0.00 | 0.12 |
| B66 | m | neutral | 0.00 | 0.00 | 0.00 | 0.00 | 0.09 | 0.43 | 0.39 | 1.07 | 0.00 | 0.00 | 0.00 | 0.00 | 0.30 |
| B67 | m | neutral | 0.03 | 0.22 | 0.00 | 0.00 | 0.02 | 0.20 | 0.46 | 1.06 | 0.00 | 0.00 | 0.06 | 0.42 | 0.35 |
| B68 | m | neutral | 0.00 | 0.00 | 0.00 | 0.00 | 0.07 | 0.29 | 0.03 | 0.30 | 0.00 | 0.00 | 0.00 | 0.00 | 0.04 |
| B69 | f | neutral | 0.03 | 0.31 | 0.00 | 0.00 | 0.03 | 0.23 | 0.18 | 0.67 | 0.00 | 0.00 | 0.00 | 0.00 | 0.12 |
| B70 | f | neutral | 0.04 | 0.32 | 0.00 | 0.00 | 0.00 | 0.00 | 0.41 | 0.96 | 0.00 | 0.00 | 0.00 | 0.00 | 0.37 |
| B71 | f | neutral | 0.03 | 0.31 | 0.00 | 0.00 | 0.15 | 0.60 | 0.12 | 0.56 | 0.00 | 0.00 | 0.00 | 0.00 | 0.00 |
| B72 | f | neutral | 0.00 | 0.00 | 0.00 | 0.00 | 0.14 | 0.52 | 0.00 | 0.00 | 0.00 | 0.00 | 0.00 | 0.00 | 0.14 |
| B73 | f | neutral | 0.00 | 0.00 | 0.00 | 0.00 | 0.01 | 0.10 | 0.20 | 0.69 | 0.00 | 0.00 | 0.00 | 0.00 | 0.19 |
| B74 | f | neutral | 0.27 | 0.90 | 0.00 | 0.00 | 0.01 | 0.10 | 0.48 | 1.10 | 0.00 | 0.00 | 0.00 | 0.00 | 0.20 |
| B75 | f | neutral | 0.01 | 0.10 | 0.00 | 0.00 | 0.27 | 0.82 | 0.03 | 0.30 | 0.03 | 0.30 | 0.00 | 0.00 | 0.20 |
| B76 | f | neutral | 0.03 | 0.30 | 0.02 | 0.20 | 0.00 | 0.00 | 0.24 | 0.88 | 0.00 | 0.00 | 0.08 | 0.48 | 0.11 |
| B77 | f | neutral | 0.00 | 0.00 | 0.00 | 0.00 | 0.22 | 0.61 | 0.00 | 0.00 | 0.00 | 0.00 | 0.00 | 0.00 | 0.22 |
| B78 | f | neutral | 0.02 | 0.20 | 0.00 | 0.00 | 0.00 | 0.00 | 0.11 | 0.51 | 0.00 | 0.00 | 0.04 | 0.24 | 0.05 |
| B79 | m | sadness | 0.22 | 0.95 | 0.00 | 0.00 | 0.00 | 0.00 | 2.99 | 1.31 | 0.00 | 0.00 | 0.15 | 0.62 | 2.62 |
| B80 | m | sadness | 0.19 | 0.79 | 0.00 | 0.00 | 0.06 | 0.43 | 2.51 | 1.33 | 0.00 | 0.00 | 0.23 | 0.81 | 2.03 |
| B81 | m | sadness | 0.00 | 0.00 | 0.00 | 0.00 | 0.00 | 0.00 | 2.80 | 1.48 | 0.00 | 0.00 | 0.64 | 1.34 | 2.16 |
| B82 | m | sadness | 0.13 | 0.62 | 0.05 | 0.37 | 0.00 | 0.00 | 2.76 | 1.32 | 0.02 | 0.14 | 0.02 | 0.21 | 2.54 |
| B83 | m | sadness | 0.03 | 0.31 | 0.01 | 0.10 | 0.00 | 0.00 | 3.08 | 1.26 | 0.00 | 0.00 | 0.19 | 0.66 | 2.85 |
| B84 | m | sadness | 0.00 | 0.00 | 0.00 | 0.00 | 0.00 | 0.00 | 2.20 | 1.49 | 0.02 | 0.20 | 0.46 | 1.07 | 1.72 |
| B85 | m | sadness | 0.05 | 0.36 | 0.00 | 0.00 | 0.00 | 0.00 | 2.73 | 1.31 | 0.00 | 0.00 | 0.22 | 0.82 | 2.46 |
| B86 | m | sadness | 0.03 | 0.30 | 0.00 | 0.00 | 0.00 | 0.00 | 3.68 | 0.91 | 0.00 | 0.00 | 0.00 | 0.00 | 3.65 |
| B87 | m | sadness | 0.02 | 0.20 | 0.02 | 0.20 | 0.00 | 0.00 | 3.12 | 1.25 | 0.00 | 0.00 | 0.05 | 0.26 | 3.03 |
| B88 | m | sadness | 0.18 | 0.70 | 0.00 | 0.00 | 0.00 | 0.00 | 2.18 | 1.29 | 0.00 | 0.00 | 0.12 | 0.51 | 1.88 |
| B89 | f | sadness | 0.05 | 0.37 | 0.01 | 0.10 | 0.00 | 0.00 | 2.60 | 1.42 | 0.00 | 0.00 | 0.33 | 0.86 | 2.21 |
| B90 | f | sadness | 0.00 | 0.00 | 0.00 | 0.00 | 0.00 | 0.00 | 2.56 | 1.29 | 0.00 | 0.00 | 0.33 | 1.01 | 2.23 |
| B91 | f | sadness | 0.00 | 0.00 | 0.00 | 0.00 | 0.00 | 0.00 | 2.43 | 1.45 | 0.00 | 0.00 | 0.04 | 0.41 | 2.39 |
| B92 | f | sadness | 0.27 | 0.90 | 0.02 | 0.21 | 0.00 | 0.00 | 3.94 | 1.00 | 0.00 | 0.00 | 0.22 | 0.80 | 3.43 |
| B93 | f | sadness | 0.00 | 0.00 | 0.05 | 0.34 | 0.00 | 0.00 | 2.69 | 1.28 | 0.00 | 0.00 | 0.04 | 0.32 | 2.60 |
| B94 | f | sadness | 0.05 | 0.36 | 0.00 | 0.00 | 0.00 | 0.00 | 2.71 | 1.61 | 0.03 | 0.30 | 0.82 | 1.48 | 1.81 |
| B95 | f | sadness | 0.02 | 0.20 | 0.02 | 0.20 | 0.01 | 0.10 | 1.26 | 1.35 | 0.03 | 0.30 | 0.44 | 0.94 | 0.74 |
| B96 | f | sadness | 0.07 | 0.40 | 0.00 | 0.00 | 0.00 | 0.00 | 1.38 | 1.48 | 0.03 | 0.30 | 0.84 | 1.30 | 0.44 |
| B97 | f | sadness | 0.33 | 0.96 | 0.29 | 0.92 | 0.00 | 0.00 | 2.14 | 1.59 | 0.01 | 0.10 | 0.18 | 0.59 | 1.33 |
| B98 | f | sadness | 0.03 | 0.30 | 0.04 | 0.28 | 0.00 | 0.00 | 1.90 | 1.47 | 0.00 | 0.00 | 0.46 | 1.05 | 1.37 |
| B99 | m | surprise | 0.00 | 0.00 | 0.00 | 0.00 | 1.72 | 1.92 | 0.00 | 0.00 | 3.18 | 1.62 | 0.00 | 0.00 | 1.46 |
| B100 | m | surprise | 0.00 | 0.00 | 0.00 | 0.00 | 0.48 | 1.15 | 0.00 | 0.00 | 3.41 | 1.20 | 0.00 | 0.00 | 2.93 |
| B101 | m | surprise | 1.13 | 1.66 | 0.01 | 0.10 | 0.00 | 0.00 | 0.03 | 0.31 | 2.82 | 1.64 | 0.00 | 0.00 | 1.65 |
| B102 | m | surprise | 0.01 | 0.10 | 0.00 | 0.00 | 0.84 | 1.32 | 0.00 | 0.00 | 2.40 | 1.42 | 0.00 | 0.00 | 1.55 |
| B103 | m | surprise | 0.06 | 0.43 | 0.00 | 0.00 | 0.62 | 1.38 | 0.00 | 0.00 | 3.73 | 1.28 | 0.00 | 0.00 | 3.05 |
| B104 | m | surprise | 0.00 | 0.00 | 0.07 | 0.49 | 0.16 | 0.70 | 0.00 | 0.00 | 3.63 | 1.10 | 0.00 | 0.00 | 3.40 |
| B105 | m | surprise | 0.59 | 1.32 | 0.02 | 0.20 | 0.05 | 0.36 | 0.00 | 0.00 | 2.94 | 1.60 | 0.01 | 0.10 | 2.27 |
| B106 | m | surprise | 0.25 | 0.94 | 0.04 | 0.40 | 0.96 | 1.73 | 0.00 | 0.00 | 3.59 | 1.66 | 0.00 | 0.00 | 2.34 |
| B107 | m | surprise | 0.56 | 1.37 | 0.03 | 0.30 | 0.11 | 0.54 | 0.00 | 0.00 | 4.02 | 1.38 | 0.00 | 0.00 | 332 |
| B108 | m | surprise | 0.58 | 1.26 | 0.00 | 0.00 | 0.00 | 0.00 | 0.00 | 0.00 | 3.12 | 1.43 | 0.00 | 0.00 | 2.54 |
| B109 | m | surprise | 1.28 | 1.92 | 0.11 | 0.66 | 0.00 | 0.00 | 0.00 | 0.00 | 3.58 | 1.85 | 0.00 | 0.00 | 2.19 |
| B110 | f | surprise | 1.12 | 1.72 | 0.01 | 0.10 | 0.00 | 0.00 | 0.05 | 0.37 | 3.19 | 1.71 | 0.00 | 0.00 | 2.01 |
| B111 | f | surprise | 0.94 | 1.62 | 0.00 | 0.00 | 0.04 | 0.29 | 0.00 | 0.00 | 3.38 | 1.61 | 0.00 | 0.00 | 2.40 |
| B112 | f | surprise | 1.02 | 1.70 | 0.01 | 0.10 | 0.08 | 0.52 | 0.00 | 0.00 | 3.64 | 1.76 | 0.00 | 0.00 | 2.53 |
| B113 | f | surprise | 1.84 | 1.92 | 0.05 | 0.37 | 0.00 | 0.00 | 0.00 | 0.00 | 2.68 | 1.93 | 0.00 | 0.00 | 0.79 |
| B114 | f | surprise | 0.55 | 1.18 | 0.02 | 0.21 | 0.30 | 0.31 | 0.00 | 0.00 | 1.91 | 1.52 | 0.00 | 0.00 | 1.04 |
| B115 | f | surprise | 0.62 | 1.42 | 0.02 | 0.21 | 0.00 | 0.00 | 0.02 | 0.21 | 3.35 | 1.37 | 0.00 | 0.00 | 2.69 |
| B116 | f | surprise | 0.73 | 1.37 | 0.00 | 0.00 | 0.09 | 0.45 | 0.03 | 0.22 | 2.54 | 1.53 | 0.00 | 0.00 | 1.69 |
| B117 | f | surprise | 0.62 | 1.31 | 0.00 | 0.00 | 0.01 | 0.10 | 0.00 | 0.00 | 2.81 | 1.43 | 0.00 | 0.00 | 2.18 |
| B118 | f | surprise | 0.07 | 0.50 | 0.00 | 0.00 | 0.05 | 0.30 | 0.00 | 0.00 | 3.66 | 0.78 | 0.00 | 0.00 | 3.54 |
| B119 | f | surprise | 0.21 | 0.84 | 0.04 | 0.40 | 0.30 | 1.07 | 0.00 | 0.00 | 4.35 | 1.13 | 0.00 | 0.00 | 3.80 |
| B120 | f | surprise | 0.47 | 1.21 | 0.04 | 0.40 | 0.00 | 0.00 | 0.00 | 0.00 | 3.57 | 1.28 | 0.00 | 0.00 | 3.06 |
| B121 | f | surprise | 1.98 | 1.89 | 0.10 | 0.50 | 0.00 | 0.00 | 0.00 | 0.00 | 2.27 | 1.93 | 0.00 | 0.00 | 0.19 |
| B122 | m | anger | 0.01 | 0.10 | 0.13 | 0.72 | 0.00 | 0.00 | 0.16 | 0.70 | 0.00 | 0.00 | 3.99 | 1.08 | 3.69 |
| B123 | m | anger | 0.00 | 0.00 | 0.00 | 0.00 | 0.00 | 0.00 | 1.04 | 1.54 | 0.01 | 0.10 | 2.33 | 1.51 | 1.28 |
| B124 | m | anger | 0.03 | 0.31 | 0.04 | 0.41 | 0.00 | 0.00 | 0.17 | 0.74 | 0.00 | 0.00 | 4.02 | 1.14 | 3.78 |
| B125 | m | anger | 0.04 | 0.32 | 0.03 | 0.31 | 0.05 | 0.34 | 0.79 | 1.47 | 0.02 | 0.21 | 1.78 | 1.48 | 0.85 |
| B126 | m | anger | 0.02 | 0.21 | 0.03 | 0.31 | 0.00 | 0.00 | 0.58 | 1.24 | 0.00 | 0.00 | 3.24 | 1.21 | 2.61 |
| B127 | m | anger | 0.04 | 0.28 | 0.07 | 0.50 | 0.00 | 0.00 | 0.04 | 0.40 | 0.00 | 0.00 | 3.76 | 1.10 | 3.61 |
| B128 | m | anger | 0.12 | 0.69 | 0.00 | 0.00 | 0.00 | 0.00 | 1.40 | 1.85 | 0.00 | 0.00 | 2.47 | 1.80 | 0.95 |
| B129 | m | anger | 0.00 | 0.00 | 0.00 | 0.00 | 0.00 | 0.00 | 0.31 | 0.99 | 0.00 | 0.00 | 3.13 | 1.28 | 2.82 |
| B130 | m | anger | 0.50 | 1.26 | 0.25 | 0.91 | 0.00 | 0.00 | 1.36 | 1.97 | 0.00 | 0.00 | 2.66 | 2.01 | 0.55 |
| B131 | f | anger | 0.08 | 0.58 | 0.00 | 0.00 | 0.00 | 0.00 | 0.02 | 0.21 | 0.00 | 0.00 | 3.28 | 1.20 | 3.18 |
| B132 | f | anger | 0.09 | 0.53 | 0.08 | 0.38 | 0.00 | 0.00 | 0.94 | 1.47 | 0.00 | 0.00 | 1.91 | 1.50 | 0.80 |
| B133 | f | anger | 0.00 | 0.00 | 0.28 | 0.95 | 0.00 | 0.00 | 0.03 | 0.31 | 0.03 | 0.31 | 2.88 | 1.44 | 2.54 |
| B134 | f | anger | 0.06 | 0.46 | 0.47 | 1.24 | 0.00 | 0.00 | 0.78 | 1.52 | 0.02 | 0.21 | 2.39 | 1.55 | 1.06 |
| B135 | f | anger | 0.12 | 0.65 | 0.00 | 0.00 | 0.00 | 0.00 | 0.23 | 0.92 | 0.00 | 0.00 | 3.54 | 1.06 | 3.19 |
| B136 | f | anger | 0.07 | 0.43 | 0.37 | 1.05 | 0.00 | 0.00 | 0.00 | 0.00 | 0.02 | 0.20 | 2.71 | 1.39 | 2.25 |
| B137 | f | anger | 0.05 | 0.50 | 0.02 | 0.20 | 0.00 | 0.00 | 0.00 | 0.00 | 0.00 | 0.00 | 4.49 | 0.85 | 4.42 |
| B138 | f | anger | 0.29 | 1.01 | 0.22 | 0.84 | 0.00 | 0.00 | 0.45 | 1.14 | 0.00 | 0.00 | 3.27 | 1.72 | 2.31 |
| B139 | f | anger | 0.00 | 0.00 | 1.12 | 1.88 | 0.00 | 0.00 | 0.06 | 0.42 | 0.00 | 0.00 | 3.08 | 1.92 | 1.90 |
| B140 | f | anger | 0.00 | 0.00 | 0.35 | 0.99 | 0.05 | 0.36 | 0.00 | 0.00 | 0.00 | 0.00 | 2.36 | 1.46 | 1.96 |
